# Organic fertilizer substitution altered the waxy maize grain quality and aroma volatiles formation by the integrated transcriptomic and metabolomic analyses

**DOI:** 10.3389/fpls.2025.1581728

**Published:** 2025-06-13

**Authors:** Xiaoqiang Zhao, Qiang Chai, Wen Yin, Hong Fan, Wei He, Cai Zhao

**Affiliations:** State Key Laboratory of Aridland Crop Science, Gansu Agricultural University, Lanzhou, China

**Keywords:** waxy maize, organic fertilizer substitution, grain quality, volatile flavors, HS-SPME-GC/MS, transcriptomics

## Abstract

Organic fertilizer substitution is an important agronomic strategy to improve waxy maize quality and aroma, while their regulatory mechanisms remain unclear. Herein, volatiles identification combined with transcriptomics to investigate grain quality and aroma formation mechanism across waxy maize “Jingkenuo 2000” at 20 days after pollination under 100% inorganic N fertilizer (IF), 100% organic N fertilizer (OF), organic fertilizer substituting 50% inorganic N fertilizer (OF_IF). Using HS-SPME-GC/MS, 20 differentially accumulated volatiles were identified, among them, volatile alkanes and esters compounds were the main volatile metabolites, accounting for 30% each, IF decreased esters metabolites (61.0%), OF increased alkanes compounds (6.5%), OF_IF had the highest esters abundance (i.e., E-2-hexenyl benzoate, hexadecanoic acid, methyl ester, octadecanoic acid, ethyl ester, octadecanoic acid, thyl ester, tetradecanoic acid, ethyl ester, and hexadecanoic acid, ethyl ester). RNA-sequencing analysis identified 1,923 unique differentially expressed genes (DEGs), and 18 core conserved DEGs among all comparison groups, which were potential candidate genes for breeding. 43 DEGs controlling sugar, amino acids, N-glycan, carotenoid, vitamin B6, and folate biosynthesis and metabolism. The interaction network analysis further revealed the complexity of quality and aroma formation. The findings provide valuable insights into grain quality and volatiles formation during waxy maize grain development under organic fertilizer substitution.

## Introduction

Waxy maize (*Zea mays* L. var. *ce*ra*tina* Kulesh), also known as sticky maize, is a special cultured type of maize that was first discovered in China in 1908 and later discovered in other regions in Asia. China has currently become the largest producer and consumer of waxy maize in the world, with a current planting area of 800,000 hectares (Luo et al., 2024). Waxy maize has more than 70% starch (consisting of nearly 100% amylopectin), more than 5% protein, 4~5% fat, and circa 2% vitamins in its endosperm (Perera et al., 2001). Due to its high nutritional contents, good flavor aroma, and convenience of processing, it is mainly consumed as a fresh-eating maize type in Asia and used in the food, textile, adhesive, and paper industries (Hao et al., 2017, 2015). In recent years, although multiple varieties of have been developed through genetic improvements, their grains yield and quality have not increased significantly (Devi et al., 2017; Yuan et al., 2022; Zhang et al., 2022). This means that the improvement of waxy maize productivity and quality is increasingly inseparable from cultivation in the future.

China has been the world’s greatest consumer of nitrogen (N) fertilizers since 1985. However, excessive use of inorganic fertilizer results in soil degradation and low use efficiency of applied fertilizers (e.g., N fertilizers) in maize cultivation, leading to considerable N losses and environmental pollution (Geng et al., 2019). Unlike inorganic fertilizers application, organic fertilizer substitutions are beneficial to soil nutrient balance, structure and moisture-holding capacity, and facilitate environmental protection (Song et al., 2020). Organic fertilizers also decrease energy consumption, greenhouse gas emissions, acidification and aquatic eutrophication (Zhang et al., 2018). The application of organic fertilizer as a partial substitute for chemical fertilizer directly alters the contents of alkali-hydrolyzed nitrogen, available potassium and phosphorus, and soil quality water content in different layers, which in turn affects the total absorption of nutrient, and potassium to varying degrees, thereby influencing the maize grain yield and biomass yield (Hou et al., 2025). Replacing 50% of synthetic fertilizer with manure was a sound option for achieving high crop yield and sustainable yield index but low chemical fertilizer under the tested cropping system (Niu et al., 2024). Meanwhile, different organic fertilizer substitutions lead to an increase of shoot dry matter, shoot nutrient uptake, grain yield, and water and N use efficiency in maize (Zhai et al., 2022; Xie et al., 2019; Moridi et al., 2019). Additionally, right N level significantly affects maize grain quality. For instance, reduction in N with right level reduced maize grain protein, soluble sugar, and starch contents, as well as the ratio of amylopectin and amylase content, while increased crude fat content compared to no-N treatment (Yu et al., 2019). With the increasing N application rate, the protein and lysine content of grains increased, while the starch content decreased (Yu et al., 2019). However, it is unclear whether rational organic fertilizer substitutions improve waxy maize grain quality and enhance aroma compared with inorganic fertilizer application. This constitutes an important knowledge gap yet to address.

Understanding changes in grain development at the molecular level will greatly aid our ability to directly and continuously improve grain quality and other aspects of maize under different environmental conditions. Previous studies have identified a limited number of genes, including *prolamin-box binding factor1* (*PBF1*; *GRMZM2G146267*) and P-loop containing nucleoside triphosphate hydrolase superfamily protein (*GRMZM2G159307*) that are involved in maize grain development and quality formation under different N applications (Ning et al., 2023; Ndlovu et al., 2022). Given the development of high-throughput sequencing technologies, such as RNA-sequencing (RNA-Seq), gene function and regulatory networks for grain quality in maize can now be elucidated at a large scale under various environmental conditions, and that knowledge can be applied for continued improvement (Lian et al., 2022). Generally, due to the difference in climate and varieties, the best harvest times of waxy maize is 20–27 days after pollination (DAP20-27), because the palatability of fresh-eating maize is the best and the accumulation of various nutrients, especially amino acid and sugars content are the highest (Niu et al., 2020). While aroma volatiles in waxy maize is unstable and the threshold value is low. With the delay of harvest time, aroma volatiles accumulation decreased and starch content increased (Chen et al., 2021). Overall, grain quality and aroma formation in waxy maize are complex, and the underlying regulatory mechanism in waxy maize under different environmental conditions remains unclear. Herein, our study used RNA-Seq analysis, head space-solid phase microextraction gas chromatography-mass spectrometry (HS-SPME-GC/MS), and quantitative real-time PCR (qRT-PCR) to investigate grain quality-related gene expression levels and volatile flavors accumulations in grains of “Jingkenuo 2000” waxy maize variety at DAP20 under three N treatments, including 100% inorganic N fertilizer (IF), 100% organic N fertilizer (OF), and organic fertilizer substituting 50% inorganic N fertilizer (OF_IF) treatments. This study will provide valuable insights into the grain quality and aroma formation mechanism in waxy maize under organic fertilizer substitutions.

## Materials and methods

### Plant materials and experimental design

The waxy maize variety “Jingkenuo 2000” was derived from the “Jingnuo6 × BN2” cross from the Beijing Academy of Agriculture and Forestry Sciences, and planted at the Oasis Agricultural Trial Station (37°30′N, 103°5′E; 1,776 m altitude; aridisol) of Gansu Agricultural University, Gansu, China, in 2022, which is located in the eastern part of the Hexi Corridor of northwestern China. At the experimental site, the annual air temperature was 7.2°C, the annual ≥ 10°C active accumulated temperature was 2,985°C, the average annual sunshine duration was 2,945 h, the annual evaporation capacities was 2,500 mm, and the annual frost-free days was 156 d, respectively. Meanwhile, the mean rainfall at the experimental site during the maize growing seasons (from April to September) was 35.6 mm and the mean temperature was 18.6°C (**Supplementary Figure 1**). Before the experiment, the depth of 0–30 cm soil was collected for basic properties test (0.94 g/kg total N and 11.7 g/kg soil organic matter).

A randomized block design was conducted with three treatments: 100% inorganic N fertilizer (IF; i.e., urea (N 45%), potassium oxide (K_2_O 60%), and phosphorus pentoxide (P_2_O_5_ 16%)), 100% organic N fertilizer (OF; i.e., amino acid organic fertilizer; the N, P_2_O_5_ and K_2_O in OF was 7.1%, 2.8%, and 3.7%, respectively), and organic fertilizer substituting 50% inorganic N fertilizer (IF_OF). Each plot was 100.0 m^2^, and the distance between plots was 2.0 m. In total, each treatment had 300 kg N/hm^2^, 150 kg P_2_O_5_/hm^2^, and 150 kg K_2_O/hm^2^, with three replications. A plastic film (0.01 mm thick, 140 cm wide) was laid out by hand over the fields and covered the soil surface, and other managements unified as field. Then, “Jingkenuo 2000” seeds were planted on 18 April 2022 at a density of 75,000 plants/hm^2^ with 0.4 m row spacing, ensuring 750 plants in each plot. At DAP20, 24 plants were selected randomly in each plot, their top ears were harvested, and the grains from the middle of 3 ears were mixed equally as a composite sample. A total of 24-grain samples in three treatments were collected, frozen in liquid nitrogen, and stored at -80°C for subsequent RNA-Seq, gene expression, and volatile flavors analyses.

### Preparation and HS-SPME-GC/MS detection of volatile flavors

Frozen grains of waxy maize “Jingkenuo 2000” at DAP20 under three N treatments (five biological replicates for each treatment) were ground to powder with liquid nitrogen, then a 3.0 g sample was transferred to a sample bottle with a 100 mL headspace. Subsequently, 15 mL saturated NaCl solution was added, and the bottle was capped and immediately placed in a 50°C constant-temperature magnetic stirrer for equilibration for 20 min. A 65 μM PDMS/DVB extraction head (Merck, USA) was inserted for headspace extraction for 30 min with oscillation at 250 rpm. After extraction, the extraction head was immediately inserted into the GC-MS injection port and analyzed at 250°C for 5 min. Further analysis was performed using a Thermo Trace 1300-ISQ7000 GC-MS (Thermo, USA). The chromatographic separation conditions were as follows: injection temperature, 260°C; carrier gas, helium (99.999%); gas flow rate, 1.0 mL/min; column temperature, maintained at 40°C for 5 min, then increased to 220°C at 5°C/min and 250°C at 20°C/min; holding time, 15 min; ion source temperature, 230°C; ionization mode, EI+ (70ev); mass range, 20~550 (NIST 2017 Spectral library). The volatile flavors were quantified by the internal standard method with 500 ng of internal standard (3-heptanone, 2-methyl-; CAS No: 13019-20-0; Sigma-Aldrich, USA). The raw data of volatile flavors were filtered and normalized to obtain relatively quantitative values of corresponding volatile flavors, then were subjected to principal component analysis (PCA) using the SIMCA v.14.0 (https://umetrics-simca.software.informer.com/). Using KEGG compound (https://www.kegg.jp/keg/compound), EMMDB (https://www.maizemdb.site/home/), and HMDB 4.0 (https://hmdb.ca/) databases to annotate metabolites. The supervised multivariate method partial least squares-discriminant analysis (PLS-DA) was used to maximize the metabolome differences between groups. The relative importance of each metabolite to the PLS-DA model was checked using the parameter variable importance in the projection (VIP). Metabolites were considered differentially accumulated metabolites (DAMs) for group discrimination with the criteria of a *P* value < 0.05 and VIP > 1 (Saccenti et al., 2014). Each DAM odor was queried using an online tool (http://www.flavornet.org/flavornet.html). Pearson’s correlation analysis among DAMs was analyzed by the GenesCloud tool (https://www.genescloud.cn) to reveal their internal relationships (Zhao et al., 2024). The chemical structure of each DAM used in the present analysis was drawn using ChemDraw Professional 15.1 Wizard (CambridgeSoft, Australia).

### RNA extraction and illumina sequencing

The total RNA from the grains of “Jingkenuo 2000” at DAP20 under different N treatments was extracted using the TransZol Plant RNA purification kit (TransGen Biotech, China). RNA purity (OD_260/280_ and OD_260/230_) was determined using the NanoDrop™ 8000 (Thermo Fisher, MA, USA), and RNA concentration was measured using a Qubit (Life Technologies, CA, USA) (**Supplementary Table 1**). The RNA integrity was assessed using an Agilent 2100 Bioanalyzer system (Agilent Technologies, CA, USA). The RNA-Seq libraries were then generated using the NEBNext Ultra RNA Library Prep Kit for Illumina according to the manufacturer’s instructions (New England Biolabs) and sequenced using the Illumina NovaSeq PE150 Sequencer at Nanjing Genepioneer Biotechnologies Company, Nanjing, China. The original raw reads were generated after excluding low-quality reads and adapter sequences using fastp. The unique reads were then aligned to the *Zea mays* B73_V4 reference genome (ftp://ftp.ensemblgenomes.org/pub/plants/release-6/fasta/zea_mays/dna/) using the HISAT v.2.2.1 (http://ccb.jhu.edu/softwre/hisat2) with default parameters (Kim et al., 2015). The data were analyzed using the HTSeq software (http://htseq.readthedocs.io/en/release_0.9.1/) based on the readcount data obtained from expression profiling. The fragments per kilobase of transcript per million mapped read (FPKM) profiles and the violin map of all genes and their gene expression levels under different N treatments were compared. The average of three replicate data in each treatment was used as the final FPKM. The differentially expressed genes (DEGs) were analyzed using DESeq R package v.1.18.0 (http://bioconductor.org/packages/release/bioc/html/DESeq.html). The DEGs between the two treatments were screened according to a false discovery rate (FDR) < 0.05 and |log_2_ FoldChange (FC)| > 1. DEGs were performed GO enrichment (http://bioinfo.cau.edu.cn/agriGO/), KEGG analysis (http://www.genome.jp/kegg/), and Nr annotation (http://www.ncbi.nlm.nih.gov/pubmed). The interaction network mapping of DEGs was analyzed by Cytoscape 3.8.2 (https://cytoscape.org/).

### Joint pearson correlation analysis between HS-SPME-GC/MS and RNA-Seq

The relative quantitative values of volatile flavors accumulation and FPKM expression level of DEGs from the grains of “Jingkenuo 2000” at DAP20 under three N treatments were transformed and normalized with log10 function and performed, their Pearson correlation using the GenesCloud tool (https://www.genescloud.cn).

### qRT-PCR analysis

The 0.5 μg purified total RNA from the grains of “Jingkenuo 2000” at DAP20 under three N treatments was reverse-transcribed to synthesize corresponding first-strand cDNA using the cDNA synthesis kit (PrimeScript™ RT Master Mix, TaKara, Japan) according to the manufacturer’s protocol. The qRT-PCR was carried out on a SuperReal PreMix Plus (SYBR Green) (Tiangen, Shanghai, China) using TransStart Tip Green qPCR SuperMix (Tran, Beijing, China) according to the manufacturer’s protocol. In the qRT-PCR reaction system, cDNA was firstly predenaturated at 95°C for 5 min, and the condition of 40 cycles of PCR amplified reaction was then set to 95°C denaturation for 10 s, 60°C annealing for 30 s, and 72°C extension for 30 s. The primer sequences of eight random genes were designed using Primer3web v.4.1.0 (https://primer3.ut.ee/) (**Supplementary Table 2**). The candidate genes relative expression levels were estimated using the 2^-△△Ct^ method, with *ZmActin1* serving as the endogenous control for normalization (Zhao et al., 2022). The correlation relationship and analysis of variance (ANOVA) among all candidate genes relative expression levels of the grains of “Jingkenuo 2000” at DAP20 under three N treatments were performed using IBM-SPSS Statistics v. 19.0 (SPSS Inc., Chicago, IL, USA).

## Results

### Volatile flavor accumulation under organic fertilizer substitution treatment

**Figure 1:**
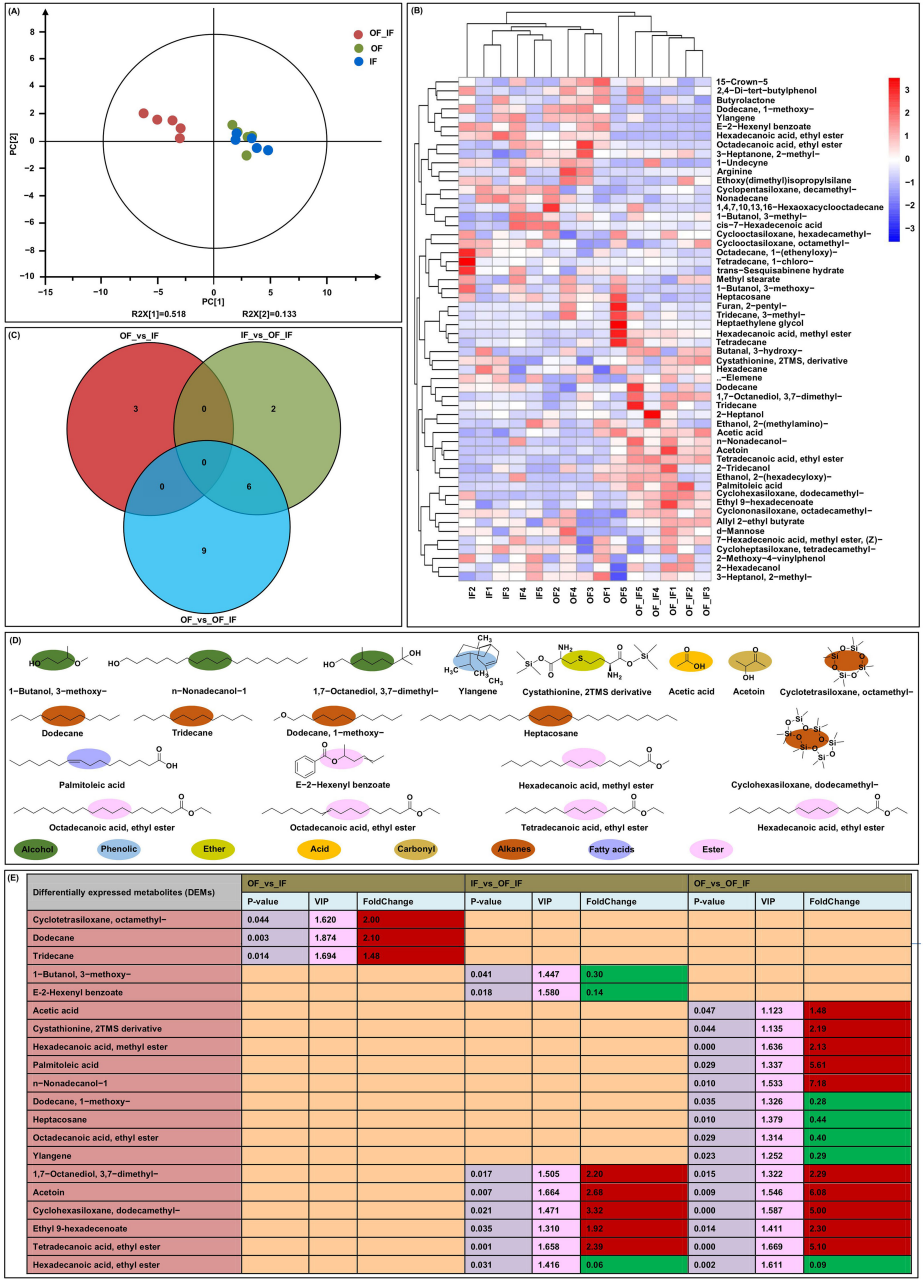
Detection of volatile flavors in grains of “Jingkenuo 2000” at 15 days after pollination under three N treatments (IF: 100% inorganic N fertilizer, OF: 100% organic N fertilizer, OF_IF: organic fertilizer substituting 50% inorganic N fertilizer) using head space-solidphase microextraction gas chromatography-mass spectrometry (HS-SPME-GC/MS). **(A)** Principal component analysis (PCA) of all volatile flavors. **(B)** A Heatmap of 56 detected volatile flavors in three N treatments. **(C)** Venn diagrams showing the number of differentially accumulated volatile flavors among three groups. **(D)** Chemical structures and classification of differentially accumulated volatile flavors. **(E)** Differentially accumulated volatile flavors were analyzed among all groups. VIP stands for variable importance in the projection. The red and green boxes represent up- and down-accumulated volatile flavors.

Soils from three N treatments were collected to reveal the effects of organic N fertilizer substitution on the aroma of grain samples in “Jingkenuo 2000” at DAP20. The PCA scores for samples exhibited a good fit (R^2^ = 0.651) and good predictive value (Q^2^ = 0.361) in the PCA scoring diagram (**Figure 1A**). The significant principal component 1 (PC1) contained 51.8% of the variables, and the significant PC2 contained 13.3% of the variables. The data showed that the distribution of samples was roughly the same and that there was no sample out of the scoring chart with Hotelling’s T2 95% confidence interval. A total of 56 volatile flavors were detected in this study (**Figure 1B**; **Supplementary Table 3**). The metabolites with VIP > 1 and *P* value < 0.05 as criterion to identified DAMs. A total of 20 unique DAMs were identified in the OF_vs_IF, IF_vs_OF_IF, and OF_vs_OF_IF groups (**Figures 1C–E**).

Of the identified DAMs, three, cyclotetrasiloxane, octamethy- (2.00-fold, had odorlessness), dodecane (2.10-fold, had petrol smell), and tridecane (1.48-fold, had malt aroma), which are alkanes, were up-accumulated in the OF_vs_IF group (**Figures 1D, E**). The finding showed that compared with IF treatment, the OF application can significantly increase alkane accumulations in waxy maize grains and the olfactory experience of waxy maize may be improved. Thereby alkanes are the most abundant volatiles in waxy maize grains under OF treatment. There were three down-accumulated DAMs in the IF_vs_OF_IF group, including alcohol, 1-butanol, 3-3-methoxyl- (0.30-fold, had pungent smell), two esters (i.e., E-2-hexenyl benzoate (0.14-fold, had fruity aroma) and hexadecanoic acid), and ethyl ester (0.06-fold, had a fruity and creamy aroma) (**Figures 1D, E**). The result indicated that the down-accumulated esters may be the major flavor volatiles in waxy maize under IF treatment compared to OF_IF treatment, resulting in the reduction of fruit aroma of waxy maize under IF treatment. Similar to the IF_vs_OF_IF group, hexadecanoic acid, ethyl ester (0.09-fold, had fruity flavor) that was down-accumulated in the OF_vs_OF_IF group. The other up-accumulated five DAMs were also simultaneously identified in IF_vs_OF_IF and OF_vs_OF_IF groups, including an alcohol of 1,7-octanediol, 3,7-dimethyl- (2.20- and 2.29-fold, had cherry and citrus flavors), a carbonyl of acetoin (2.68- and 6.08-fold, had a creamy aroma), an alkane of cyclohexasiloxane, dodecamethyl- (3.32- and 5.00-fold, had odorlessness), two esters of ethyl-9-hexadecenoate (1.92- and 2.30-fold, had apple and banana acidity) and tetradecanoic acid, and ethyl ester (2.39- and 5.10-fold, had fragrant iris oil aroma) (**Figures 1D, E**). The results determined that these six core flavor volatiles may be marker metabolites of waxy maize under OF_IF treatment, and have higher contribution for the waxy maize aroma under organic fertilizer substitution treatment. Additionally, in the OF_vs_OF_IF group, five up-accumulated DAMs were also identified, including an acid of acetic acid (1.48-fold, had pungent smell of vinegar), an ether of cystathionine, 2TMS derivative (2.19-fold, had pungent smell), an ester of hexadecanoic acid, methyl ester (2.13-fold, had waxy smell), an fatty acid of palmitoleic acid (5.61-fold, had special smell), and an alcohol of n-nonadecanol-1 (7.18-fold, had *Neotinea ustulata* aroma). Four down-accumulated DAMs were also identified, including two alkanes of dodecane, 1-methoxy- (0.28-fold, had wood and ambergris aroma) and heptacosane (0.44-fold), an ester of octadecanoic acid, ethyl ester (0.40-fold, had odorlessness), and a phenolic of ylangene (0.29-fold, had clove aroma) (**Figures 1D, E**). There were more flavor volatiles, showing varied accumulation profiles in the OF_vs_OF_IF group than the others. Moreover, the Pearson correlation showed that 74 pairs of flavor volatiles with significant (*p* < 0.05) correlation among the three groups (**Supplementary Figure 2**), indicating that these volatile flavors under different N treatments form a highly interconnected network, altering the aroma and taste of waxy maize.

### RNA-Seq data quality

**Figure 2:**
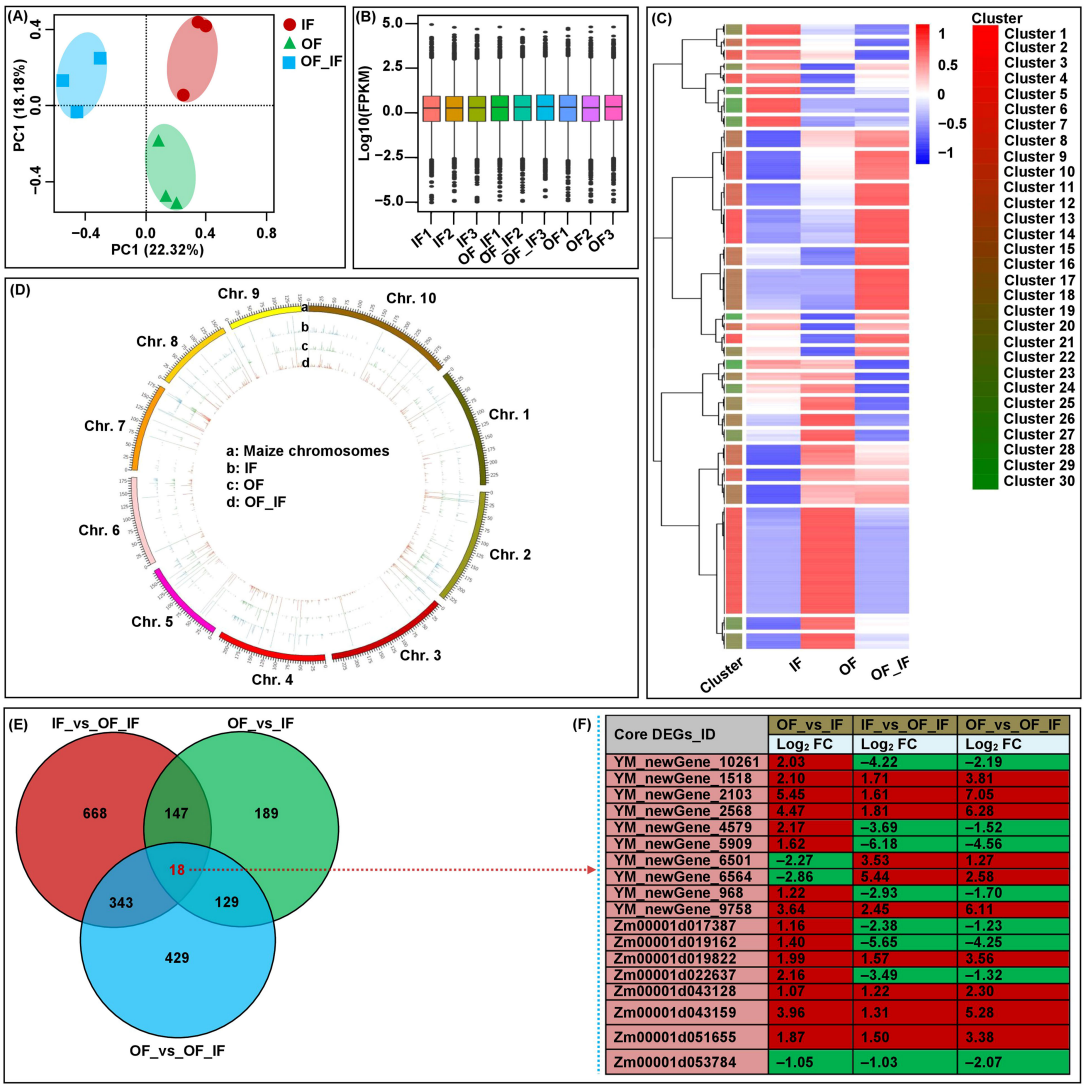
Global transcriptome sequencing and differentially expressed genes (DEGs) analysis in grains of “Jingkenuo 2000” at 15 days after pollination under three N treatments (IF: 100% inorganic N fertilizer, OF: 100% organic N fertilizer, OF_IF: organic fertilizer substituting 50% inorganic N fertilizer). **(A)** Principal component analysis (PCA) of RNA-sequencing data. **(B)** Gene expression in all samples. Boxplots with different colors indicate different samples analyzed at regular intervals. **(C)** Cluster analysis of DEGs based on gene expression in all samples. **(D)** Chromosome-wide distribution and expression profiles of all expressed genes across all samples. **(E)** Venn diagrams showing the number of DEGs and core conserved DEGs across three groups. **(F)** The expression levels of 18 core conserved DEGs in all groups. The red and green boxes represent up- and down-regulated DEGs, respectively. The log_2_FC value is the log2 fold-change of DEGs.

To explore the patterns of the changes in the expression of genes in waxy grains at DAP20 under three treatments, RNA-Seq was performed on nine libraries of grains of the “Jingkenuo2000” variety under the IF, OF, and IF_OF treatments (three biological replicates for each sample). An average of 8.05 G clean data were obtained from each sample, the Q30 value exceeded 91% and the GC content distribution was 53.54~54.33% (**Supplementary Table 4**). After filtering low-quality reads, 90.24~92.15% of clean reads were mapped to the *Zea mays* B73_V4 reference genome (**Supplementary Table 4**). PCA of the RNA-Seq datasets for all samples showed that transcriptomes from the three N treatments were clearly separated into three groups, and their replicates were closely clustered (**Figure 2A**). The findings indicated that the genes were differentially expressed in waxy maize grains in response to different N treatments, and the data in good merits furthering demonstrating that the identified DEGs were induced by different N treatments (**Figure 2A**).

**Figure 3:**
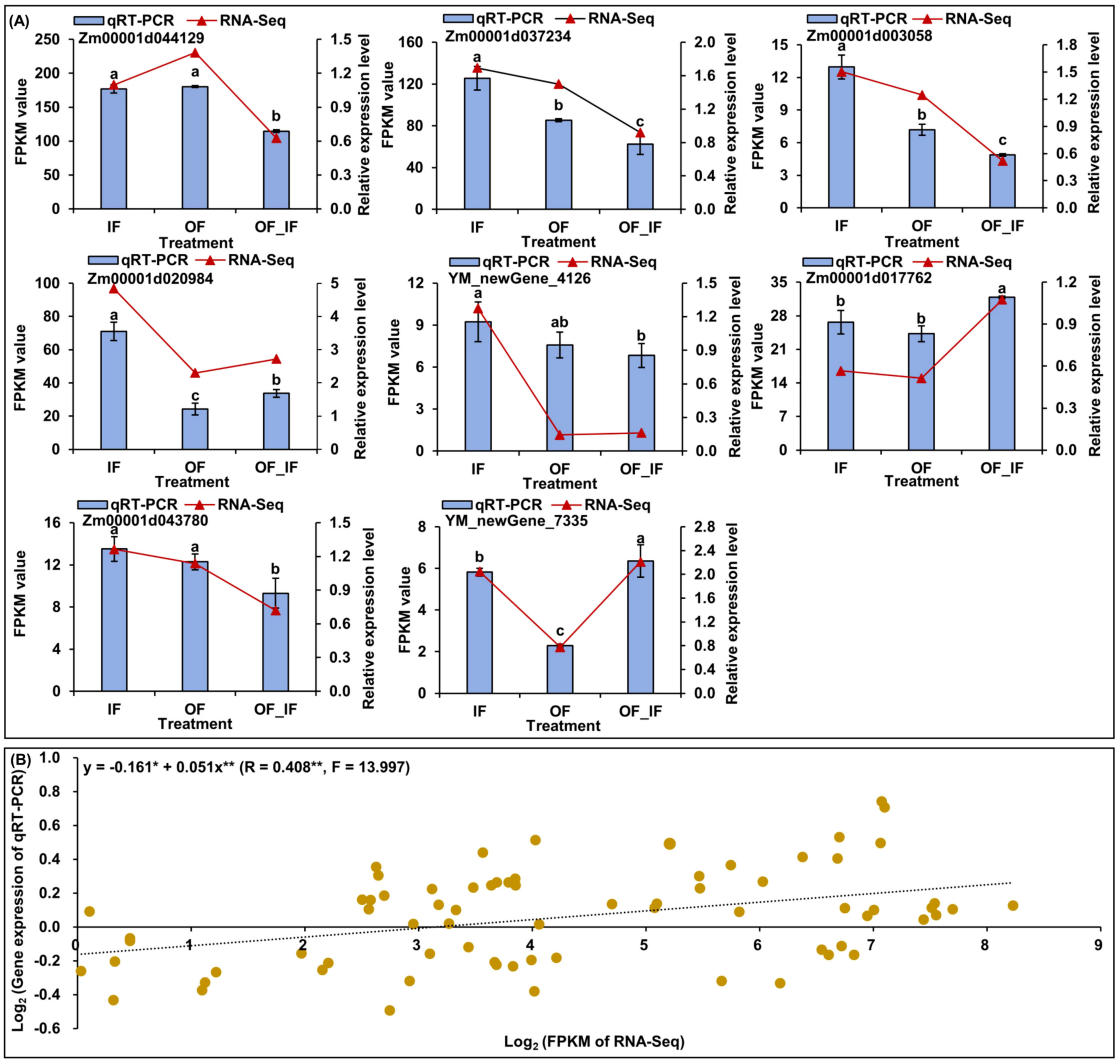
The quantitative real-time PCR (qRT-PCR) expression analysis of eight random genes in grains of “Jingkenuo 2000” at 15 days after pollination under three N treatments (IF: 100% inorganic N fertilizer, OF: 100% organic N fertilizer, OF_IF: organic fertilizer substituting 50% inorganic N fertilizer). **(A)** Comparisons of the changes in relative expression levels of eight genes by qRT-PCR and RNA-sequencing (RNA-Seq). Different lowercase letters of qRT-PCR gene expression levels under different N treatments indicate significant differences at *p* < 0.05, ANOVA. **(B)** The correlation between qRT-PCR and RNA-Seq data. ** or * indicates a significant correlation with *p* < 0.01 or *p* < 0.05, respectively.

To further verify the reliability of RNA-Seq data and provide valuable gene resources for improving waxy maize quality, we analyzed and found differences (*p* < 0.05) in the relative expression levels of eight random genes in “Jingkenuo 2000” grains at DAP20 under three N treatments using qRT-PCR (**Figure 3A**). The genes showed similar expression levels of up- or down-regulation based on the expression patterns from qRT-PCR and RNA-Seq analysis (**Figure 3A**), and there was a good linear relationship between RNA-Seq dataset and qRT-PCR expression levels (y=-0.161*+0.051x**; R=0.408**, F=13.997) (**Figure 3B**).

### DEGs and core conserved degs identification

A total of 40,789 expressed genes were identified, including 4,824 novel genes (**Figures 2C, D**). Based on the criteria FDR < 0.05 and |log_2_ FC| > 1, a total of 1,923 unique DEGs were identified among three groups, varying from 483 (OF_vs_IF) to 1,176 (IF_vs_OF_IF) DEGs (**Figure 2E**). The abundant expression levels of these DEGs under different N treatments could affect the grain development and quality formation of waxy maize at DAP20, in particular, the largest number (1,176) of DEGs were identified in the IF_vs_OF_IF group. We postulated that such core conserved DEGs may be associated with grain development and quality formation in the “Jingkenuo2000” variety under diverse fertilization treatments.

Fortunately, 18 core conserved DEGs were found among all groups (**Figures 2E, F**; **Supplementary Table 5**). Specifically, *Zm00001d019162* has nutrient reservoir activity (GO: 0045735) and may be positively involved in the accumulation of nutrients during endosperm development in waxy maize under OF_IF treatment. *YM_newGene_4579*, encoding a vegetative cell wall protein, was a structural constituent of the cell wall (GO: 0005199) and regulates the thickness of corn husk. The protein (de)ubiquitination is essential in regulating many cellular processes and modulating diverse interactions in maize grain development (Fan et al., 2021). *YM_newGene_6501* (ubiquitin carboxyl-terminal hydrolase 8), with thiol-dependent ubiquitin-specific protease activity (GO: 0004843), involves in ubiquitin-dependent protein catabolic processes (GO: 0006511) and protein deubiquitination (GO: 0016579). *YM_newGene_9758* and *YM_newGene_2568* has phosphoprotein phosphatase activity (GO: 0004721), which may play roles in signal transduction in response to maize N supply. *Zm00001d053784* and *Zm00001d043128* are involved in defense responses to fungi (GO: 0050832) and defense responses (GO: 0006952), respectively. *Zm00001d043159*, *YM_newGene_6564*, and *YM_newGene_2103* have transcriptional activator activity, RNA polymerase II proximal promoter sequence-specific DNA binding (GO: 0001077), and sequence-specific DNA binding (GO: 0043565), respectively.

### Grain quality formation key pathways identification under organic fertilizer substitution

Many developmental processes and metabolic pathways of DEGs were explored by GO terms and top 20 KEGG pathways (**Supplementary Figures 3A, B**). These processes are vital for maize grain development under various N conditions, which were complex and likely regulated by the interaction networks consisting of multiple pathways, such as “starch and sucrose metabolism (map00500)”, “glycine, serine and threonine metabolism (map00260)”, “N-glycan biosynthesis (map00510)”, “carotenoid biosynthesis (map00906)”, and “folate biosynthesis (map00790)”, which are related to “cellular process”, “metabolic process”, and “response to stimulus” (**Supplementary Figures 3A, B**).

### Starch and sucrose metabolism under organic fertilizer substitution

**Figure 4:**
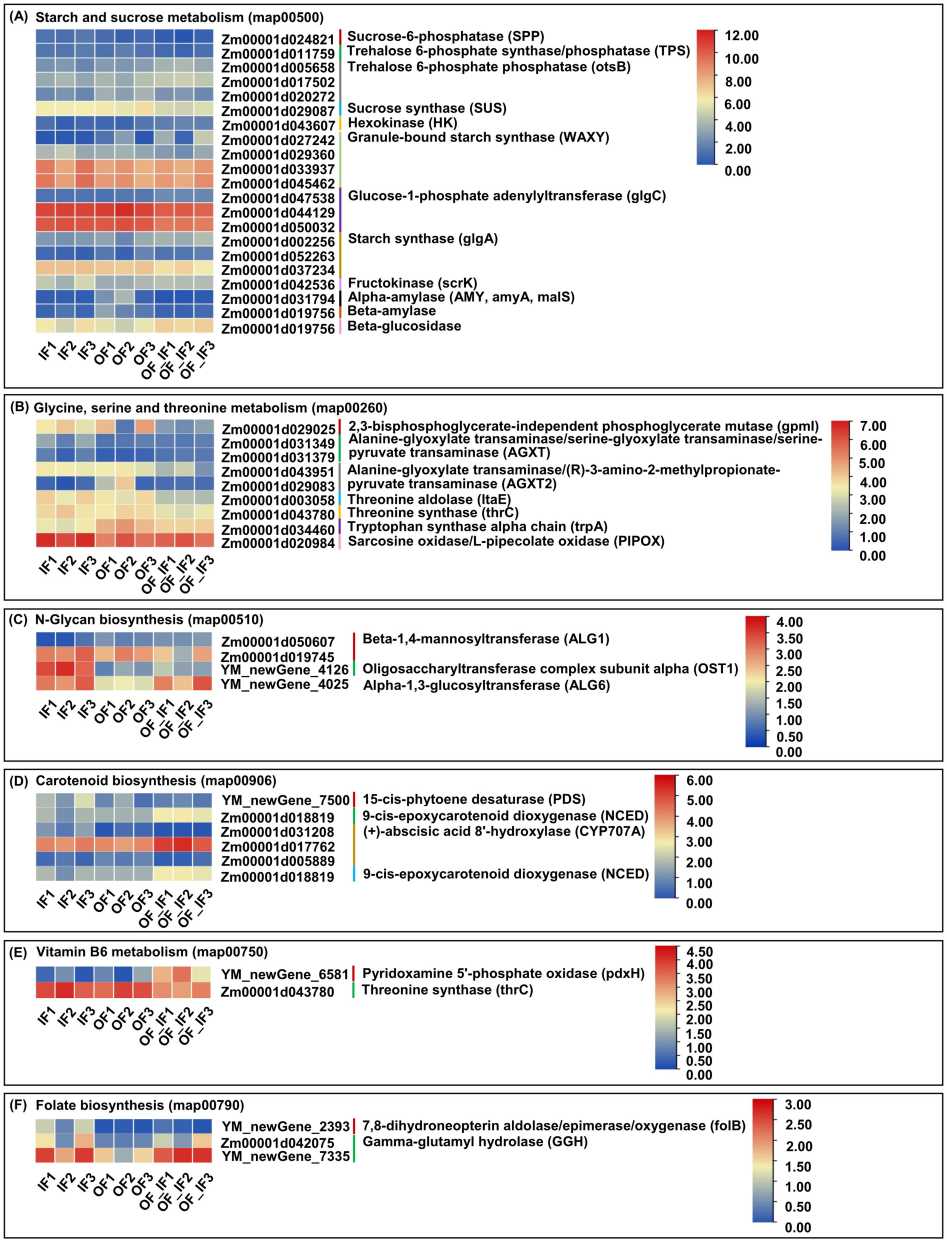
The fragments per kilobase of transcript per million mapped read (FPKM) expression profiles of differentially expressed genes (DEGs) in grains of “Jingkenuo 2000” at 15 days after pollination under three N treatments (IF: 100% inorganic N fertilizer, OF: 100% organic N fertilizer, OF_IF: organic fertilizer substituting 50% inorganic N fertilizer). Values indicate the log2 (FPKM+1). **(A)** DEGs involved in starch and sucrose metabolism. **(B)** DEGs involved in glycine, serine and threonine metabolism. **(C)** DEGs involved in N-glycan biosynthesis. **(D)** DEGs involved in carotenoid biosynthesis. **(E)** DEGs involved in vitamin B6 metabolism. **(F)** DEGs involved in folate biosynthesis.

Starch is an important accumulated storage carbohydrate during maize grain development, and thus, the starch and sucrose metabolism (map00500) pathway could be an important target of regulation (Ma et al., 2019). In this study, we identified 21 unique DEGs involved in starch and sucrose metabolism among the three comparison groups (**Figure 4A**; **Supplementary Table 6**). Overall, *Zm00001d024821* (2.77-fold), encoding sucrose-6-phosphatase (SPP), was identified in the IF_vs_OF_IF group, and *Zm00001d029087* (1.07-fold and 1.11-fold), encoding sucrose synthase (SUS), was identified in both the IF_vs_OF_IF and OF_vs_OF_IF groups, showing significantly down-regulated expression, influencing sucrose synthesis. In the IF_vs_OF_IF group, only one trehalose 6-phosphate synthase/phosphatase (TPS) gene, *Zm00001d011759* (1.09-fold), displayed significantly down-regulated expression; however, two trehalose 6-phosphate phosphatase (otsB) genes, *Zm00001d005658* (1.28-fold) and *Zm00001d017502* (1.04-fold), showed significantly up-regulated expression. Conversely, only one otsB gene, *Zm00001d020272*, was downregulated in both the OF_vs_IF (1.32-fold) and OF_vs_OF_IF (1.20-fold) groups. These findings indicated that trehalose synthesis genes showed varied expression levels under different N treatments. Only one hexokinase (HK), encoded by the *Zm00001d043607* gene, was significantly up-regulated in both the IF_vs_OF_IF (1.51-fold) and OF_vs_OF_IF (1.30-fold) groups, and only one fructokinase (scrK), encoded by *Zm00001d042536* (1.11-fold), was also significantly up-regulated in the OF_vs_IF group, therefore playing roles in the pathways involved in the metabolism of sugars (fructose) and mannose metabolism (starch and sucrose metabolism). In addition, two types of starch, i.e., the UDP-glucose (UDP-G) substrates, were catalyzed by granule-bound starch synthase (WAXY) to produce amylose; consequently, two down-regulated WAXY genes (*Zm00001d029360* and *Zm00001d033937*, 1.48-fold and 1.14-fold) were identified in the IF_vs_OF_IF group, and two other WAXY genes (*Zm00001d027242* and *Zm00001d045462*, 6.67- and 1.12-fold, up- and down-regulated, respectively, in the IF_vs_OF_IF group; 4.75- and 1.08-fold, down-regulated and up-regulated, respectively, in the OF_vs_IF group) had different expression patterns. A-D-Glucose-1P is commonly catalyzed by glucose-1-phosphate adenylyltransferase (glgC) and starch synthase (glgA) to produce amylopectin; similarly, three glgC genes (i.e., *Zm00001d047538*, *Zm00001d044129*, *Zm00001d050032*) were identified in both the IF_vs_OF_IF and OF_vs_IF groups, and three glgAs (i.e., *Zm00001d002256*, *Zm00001d052263*, *Zm00001d037234*) were identified in the IF_vs_OF_IF group. In conclusion, the differential expression patterns of these DEGs significantly affected starch formation under the OF and OF_IF treatments compared to the IF treatment. Subsequently, enzymes such as alpha-amylase (AMY) encoded by the *Zm00001d031794* gene (4.35- and 6.90-fold) and beta-amylase encoded by *Zm00001d019756* (2.34- and 3.08-fold) were significantly down-regulated in both the OF_vs_IF and OF_vs_OF_IF groups, resulting in the inhibition of maltose and dextrin accumulation.

### Glycine, serine and threonine metabolism under organic fertilizer substitution

Amino acids are essential bioactive molecules in plants and regulate many physiological processes, including plant growth and development and stress responses (Wang et al., 2021). At the onset of rapid grain filling, the transport rates of total soluble sugars and amino acids into the endosperm are linearly related in maize (Seebauer et al., 2010). A total of nine unique DEGs were involved in glycine, serine and threonine metabolism (map00260) in all comparison groups (**Figure 4B**; **Supplementary Table 7**). There was one 2,3-bisphosphoglycerate-independent phosphoglycerate mutase (gpmI; *Zm00001d029025*, 2.76-fold) gene in the IF_vs_OF_IF group, one tryptophan synthase alpha chain (trpA; Zm00001d034460, 1.20-fold) in the OF_vs_IF group with down-regulated expression, and two alanine-glyoxylate transaminases/serine-glyoxylate transaminases/serine-pyruvate transaminases (AGXT; *Zm00001d031349*, 1.42- and 1.48-fold, down- and up-regulated in the IF_vs_OF_IF and OF_vs_IF groups, respectively; *Zm00001d031379*, 1.39-fold, up-regulated in the OF_vs_OF_IF group) were identified in all three comparison groups. These DEGs cooperatively formed a complex regulatory network for serine synthesis in maize grains under three N treatments. In addition, one threonine aldolase (ltaE; *Zm00001d003058*, 1.71- and 1.31-fold) gene showed down-regulated expression in both the IF_vs_OF_IF and OF_vs_IF groups, and one threonine synthase (thrC; *Zm00001d043780*, 1.01-fold) displayed down-regulated expression in the IF_vs_OF_IF group, regulating threonine synthesis in maize grains under the different N treatments. In parallel, except for the above detected ltaE and two AGXT DEGs, two alanine-glyoxylate transaminase/(R)-3-amino-2-methylpropionate-pyruvate transaminase (AGXT2) genes, *Zm00001d043951* (1.53- and 1.32-fold) and *Zm00001d029083* (4.12- and 3.59-fold) were down-regulated in all comparison groups, and these DEGs worked together to regulate glycine synthesis in maize under N treatments.

### N-glycan biosynthesis under organic fertilizer substitution

N-glycosylation is a complex process that encompasses the biosynthesis and modification of sugar moieties in the endoplasmic reticulum and Golgi (Richard and Anna, 2009). Increasing evidence has shown that N-glycan production is significantly correlated with plant growth and development, such as tiler bud formation, internode and panicle development, and grain filling in rice (Harmoko et al., 2016). In this study, we identified four unique DEGs responsible for the N-glycan biosynthesis (map00510) pathway, including two beta-1,4-mannosyltransferase (ALG1; *Zm00001d050607*, 1.24-fold, up-regulated; *Zm00001d019745*, 1.19-fold, down-regulated) genes identified in the IF_vs_OF_IF group; one oligosaccharyltransferase complex subunit alpha (OST1; *YM_newGene_4126*) was down-regulated (2.92-fold) in the IF_vs_OF_IF group and up-regulated (3.30-fold) in the OF_vs_IF group; and one alpha-1,3-glucosyltransferase (ALG6; *YM_newGene_4025*) was up-regulated in the OF_vs_IF (1.53-fold) and OF_vs_OF_IF groups (**Figure 4C**; **Supplementary Table 8**). Thus, the positive or negative expression of these DEGs significantly responded to N-glycan production in maize grains under the different N treatments.

### Carotenoid biosynthesis under organic fertilizer substitution

Carotenoids are a diverse group of phytochemicals and have a broad range of biological functions (Liu, 2013). In animals, carotenoids are the precursors for vitamin A and serve as important antioxidants to prevent human diseases such as cancer, cardiovascular disease, and light-induced erythema (Paine et al., 2005). Maize is one of the essential sources of carotenoids, and the carotenoid content of sweetcorn can reach up to 1 978 μg g^-1^ fresh weight (Liu et al., 2017). In the carotenoid biosynthesis (map00906) pathway, a total of six unique DEGs, including *YM_newGene_7500* (2.07-fold), encoding 15-cis-phytoene desaturase (PDS), and *Zm00001d031208*, encoding (+)-abscisic acid 8’-hydroxylase (CYP707A), were down-regulated, and *Zm00001d018819*, encoding 9-cis-epoxycarotenoid dioxygenase (NCED) was up-regulated in the IF_vs_OF_IF group, while *Zm00001d017762* (CYP707A; 1.13-fold, upregulated), *Zm00001d005889* (CYP707A; 1.91-fold, down-regulated), and *Zm00001d018819* (NCED, 1.31-fold, up-regulated) were identified in the OF_vs_OF_IF group (**Figure 4D**; **Supplementary Table 9**). The expression levels of these corresponding DEGs changed, affecting carotenoid accumulation in maize grains among the various comparison groups.

### Vitamin B6 metabolism under organic fertilizer substitution

Vitamin B, one of the microelements, is essential for maintaining normal life activities. Instead of synthesizing vitamin B themselves, humans and animals obtain vitamin B from external sources, especially cereal products, which are among the most important staple foods (Regvar et al., 2011). Therefore, the analysis of vitamin B metabolism is significant for improving maize grains quality and human health (Azam et al., 2022). In this study, we identified two unique DEGs involved in the vitamin B6 metabolism (map00750) pathway, i.e., the *YM_newGene_6581* gene related to pyridoxamine 5’-phosphate oxidase (pdxH) was positively regulated in the IF_vs_OF_IF (3.52-fold) and OF_vs_OF_IF (2.99-fold) groups, and the *Zm00001d043780* gene related to thrC was negatively regulated in the IF_vs_OF_IF (1.01-fold) group (**Figure 4E**; **Supplementary Table 10**).

### Folate biosynthesis under organic fertilizer substitution

Folates are essential elements for human growth and development, and folate deficiency can lead to severe disorders. Waxy maize is a rich source of folates (Song et al., 2021). Therefore, understanding the molecular mechanism underlying folate biosynthesis in waxy maize will help in the development of nutritionally fortified varieties. In this study, we identified three unique DEGs involved in folate biosynthesis (map00790), i.e., *YM_newGene_2393*, controlling 7,8-dihydroneopterin aldolase/epimerase/oxygenase (folB), showed differential expression levels in the IF_vs_OF_IF (3.89-fold, down-regulated) and OF_vs_OF_IF (5.61-fold, up-regulated) groups, and two gamma-glutamyl hydrolase (GGH) genes, *Zm00001d042075* (2.35- and 1.77-fold) and *YM_newGene_7335* (1.69-fold), were up-regulated in both the OF_vs_IF and OF_vs_OF_IF groups (**Figure 4F**; **Supplementary Table 11**).

## Discussion

### Identification of key genes for quality formation of waxy maize grains under organic fertilizer substitution

Grain filling, the grain developmental stage during which proteins and carbohydrates accumulate in maize, has a marked influence on grain weight and quality and has been of great interest in maize biology and agronomy. Maize grain development was divided into early (DAP0-8), middle (DAP10-28), and late (DAP30-38) stages via maize grain dynamic transcriptome analysis (Chen et al., 2014). Previous studies have focused on the early stage of endosperm development, as it is accompanied by the most dramatic changes (Xin et al., 2013; Zhan et al., 2015). The middle stage is also of great biological and economic importance, as it corresponds to the linear phase of grain filling; the accumulation of proteins related to carbohydrate metabolism peaked approximately DAP15–20 and subsequently decreased until DAP30 (Kim et al., 2020). Waxy maize has many excellent characteristics in the food and nonfood industries, and the N application level has significant effects on its grain yield and quality (Lou et al., 2024). In this regard, in this study, we exploited “Jingkenuo2000” waxy maize grain RNA-Seq expression profiles at DAP20 under three N treatments to identify DEGs responsible for grain quality formation. In total, 1,923 unique DEGs were detected among the IF_vs_OF_IF, OF_vs_IF, and OF_vs_OF_IF groups (**Figure 2E**). The varying expression levels of these DEGs under different N treatments could affect the grain development and quality formation of waxy maize at DAP20, which was consistent with the results of Lou et al. (2024), who also reported that compared with the quantitative fertilizer nitrogen treatment, the 20%, 40%, and 60% organic fertilizer substitution treatments increased the fresh ear yield of waxy maize by 3.08%, 13.61%, and 3.20%, respectively. At the same time, the appearance and taste quality scores of waxy maize grains under the three organic fertilizer substitutions were higher than those under the quantitative fertilizer nitrogen treatment. Furthermore, the GO enrichment and top 20 KEGG pathway analyses also showed that the maize grains had evolved a range of molecular strategies that predominated from the fertilization to mature seed stages under different N treatments, and the three N comparison groups showed changes in many developmental processes and metabolic pathways. The processes were clearly complex and were likely regulated by the interaction networks consisting of multiple pathways; “starch and sucrose metabolism (map00500)”, “glycine, serine and threonine metabolism (map00260)”, “N−glycan biosynthesis (map00510)”, and “carotenoid biosynthesis (map00906)”, which are related to “cellular process”, “metabolic process”, and “response to stimulus”, may play more vital roles in waxy maize grain development under various comparison groups.

Starch is the major component of maize grains, accounting for approximately 70% of the final dry weight of grains; therefore, grain filling is mainly a process of starch synthesis (Zhang et al., 2007). The substitution of some chemical fertilizers with organic fertilizers increased the total starch and pullulan content of waxy maize grains, reduced the content of grain protein and soluble sugar, improved grain texture characteristics, increased grain hardness, elasticity and chewiness, and decreased cohesion (Lou et al., 2024). SPP catalyzes the final step in sucrose biosynthesis (Albi et al., 2016), and the activity of SPP is negatively associated with starch accumulation but is positively associated with sucrose formation. SUS is a glycosyl transferase enzyme that plays a key role in sucrose metabolism, primarily in sink tissues, which catalyzes the reversible cleavage of sucrose to fructose and either UDP-G or adenosine diphosphate glucose (ADP-G) and provides substrate and energy for starch synthesis (Ofer and David, 2019). We only detected one DEG for SPP in the IF_vs_OF_IF group and one DEG for SUS in the IF_vs_OF_IF and OF_vs_OF_IF groups (**Figure 4A**). Compared with 100% organic N fertilizer or 100% chemical N fertilizer treatment, 50% organic fertilizer substitution treatment may promote sucrose deposition in waxy maize grains. In addition, trehalose is a nonreducing disaccharide, and during trehalose biosynthesis, TPS catalyzes the formation of trehalose-6-phosphate (T6P) from UDP-G and 6-phosphate glucose, which is then catalyzed by otsB to produce trehalose. The T6P concentration was reduced, while the sucrose concentration increased, in ear spikelets, resulting in an increase in the grain set and harvest index of maize plants overexpressing otsB (Nuccio et al., 2015). One downregulated TPS DEG and three up-regulated otsB DEGs were found in the IF_vs_OF_IF group, indicating that organic fertilizer substitution at 50% could activate otsB gene expression and then promote sucrose and trehalose accumulation in waxy maize grains. Interestingly, starch consists of amylopectin and amylose. Amylopectin is synthesized by the catalytic action of several enzymes, such as ADP glucose pyrophosphorylase (AGPase), glgA, starch branching enzymes (BEs), and starch debranching enzymes (DBEs); in contrast, AGPase and WAXY are involved in amylose production (Wang et al., 2014). Our study identified four DEGs encoding WAXY in the IF_vs_OF_IF and OF_vs_IF groups and three DEGs encoding glgA in the IF_vs_OF_IF group (**Figure 4A**); in addition, three DEGs encoding glgC were also identified in both the IF_vs_OF_IF and OF_vs_IF groups and may be involved in amylopectin production (**Figure 4A**). Moreover, Chen et al. (2022) reported that multiple HK and beta-glucosidase genes involved in glycolysis, starch and sucrose metabolism were identified in developing grains of sweet corn with a genome-wide transcriptome analysis; consistent with this, we found one HK and one beta-glucosidase DEG among the multiple comparison groups (**Figure 4A**). These findings thus indicated that the molecular mechanism of starch metabolism in waxy maize grains under different N treatments was complex. Additionally, in the two comparison groups, we also identified DEGs involved in starch and sucrose metabolism in waxy maize grains under various N treatments, including one scrK, one AMY, and one beta-amylase, which remain to be studied.

Notably, N-glycans play important roles in various biological phenomena, and their structures and expression are sensitive to environmental changes. It is well known that N-glycosylation occurs in two phases, namely, core glycosylation in the endoplasmic reticulum and glycan maturation in the Golgi apparatus (Richard and Anna, 2009). In the endoplasmic reticulum, preassembled core oligosaccharides are transferred to asparagine residues of the Asn-X-Ser/Thr motifs in nascent polypeptides via the action of an OST (Schaewen et al., 2008). Phenotypic observation of the OST subunit mutant stt3a showed that protein glycosylation could affect salt tolerance and root growth of *Arabidopsis thaliana* (Koiwa et al., 2003). A series of ALGs (asparagine-linked glycosyltransferases) catalyze the transfer of glycogroups from the activated donor molecule (including monosaccharides such as glucose, fructose, and ribose; oligosaccharides such as sucrose and lactose, linked by 2–10 glucoside bonds; polysaccharides such as starch, glycogen and cellulose, linked by more than 10 glucoside bonds) to the receptor molecule (Lim and Bowles, 2004). In our study, four DEGs related to two ALG1s, one ALG6, and one OST1 were mapped among the three comparison groups (**Figure 4C**). Therefore, these DEGs of the N-glycan biosynthesis pathway may control the formation of maize grain quality under different N treatments.

Maize grain development depends on the availability of carbon and nitrogen assimilates supplied by plants and the capacity of the grain to use them. In a certain nitrogen application range, the contents of amino acids in maize grains increased with increasing nitrogen application, and the contents of amino acids in maize grains were greatly affected and varied (Peng et al., 2021). Zhang et al. (2005) reported that the variation in glycine, serine and threonine contents in grains was affected by various factors, such as maize variety and nitrogen supply level. In the present study, we further identified the DEGs involved in glycine, serine and threonine metabolism. ltaE catalyzed the interconversion of threonine and glycine plus acetaldehyde in a pyridoxal phosphate-dependent manner (Liu et al., 2015); thrC was the last enzyme in the threonine pathway, and *Arabidopsis* plants expressing thrC in the sense orientation showed a higher level of threonine; pipecolate was the endogenous substrate for PIPOX in *Arabidopsis* plants, and when the PIPOX gene was expressed at a low level, *Arabidopsis* plantlets slowly metabolized the supplied [(14)C] sarcosine to glycine and serine (Goyer et al., 2004). Our results showed that multiple DEGs involved in glycine, serine and threonine metabolism pathways were identified among all comparison groups, including the one down-regulated pgpmI gene in the IF_vs_OF_IF group, one down-regulated trpA gene in the OF_vs_IF group, two AGXT genes in three comparison groups, one down-regulated ltaE gene in both the IF_vs_OF_IF and OF_vs_IF groups, one down-regulated thrC gene in the IF_vs_OF_IF group, and two down-regulated AGXT2 genes in all comparison groups (**Figure 4B**). Therefore, these identified DEGs constituted a very complex interaction network, and they interacted with each other to maintain a dynamic balance among glycine, serine, and threonine levels in waxy maize grains under different N supplies.

Due to the nutritional and commercial value of carotenoids, the carotenoid biosynthesis pathway in plants has been determined. PDS is an essential enzyme for carotenoid synthesis, using the CRISPR–Cas9 system to examine the activity of downstream genes involved in the biosynthesis of carotenoids showed that the contents of carotenoids and chlorophylls in the leaf tissue of nine *NtPDS Nicotiana tabacum* transgenic lines were reduced (Nezhdanova et al., 2023). We detected one down-regulated DEG for PDS in the IF_vs_OF_IF group (**Figure 4D**). This indicates that 100% chemical N fertilizer treatment mediated specific PDS gene negative expression, which was not conducive to carotenoid formation in waxy maize grains. Hydroxylation is a pivotal step in converting carotene to xanthophyll via b-ring hydroxylase and e-ring hydroxylase (Liu et al., 2017). Wang et al. (2023) identified a spontaneous cucumber mutant with yellow flesh (*yf-343*) that accumulated more β-cryptoxanthin and less lutein than regularly cultivated European glasshouse-type cucumbers. The candidate gene encoding CYP707A was further identified through fine mapping and gene sequencing. This study also identified one down-regulated DEG encoding CYP707A in the IF_vs_OF_IF group (**Figure 4D**); this gene may promote β-cryptoxanthin and xanthophyll accumulation under 50% organic fertilizer substitution. The difference was the production of xanthoxin through the oxidative cleavage of 9-cisepoxyxanthophylls by NCED. We also identified two DEGs for NCED that were positively regulated in the IF_vs_OF_IF and OF_vs_OF_IF groups (**Figure 4D**).

Vitamin B6 is an essential co-factor for a range of biochemical reactions; although plants are a major source of vitamin B6 in the human diet, our understanding of the pathway therein is very limited. In this study, we only identified two DEGs involved in the vitamin B6 metabolism pathway: one pdxH gene was up-regulated in the IF_vs_OF_IF and OF_vs_OF_IF groups, and one thrC gene was down-regulated in the IF_vs_OF_IF group (**Figure 4E**). This result could provide new insights into vitamin B6 metabolism in maize grains under different nitrogen supplies. In addition, the level of the B6 vitamer pyridoxal 5′-phosphate (PLP) was drastically reduced in both the embryo and endosperm of the *small kernel2* (*smk2*) mutant of maize, whereas embryogenesis of the *smk2* mutant was arrested at the transition stage and endosperm formation was nearly normal; further cloning revealed that *Smk2* encodes the glutaminase subunit of the PLP synthase complex involved in vitamin B6 biosynthesis, indicating that vitamin B6 has differential effects on embryogenesis and endosperm development in maize (Yang et al., 2017). Moreover, folate, a bioactive complex of vitamin B, is essential for DNA synthesis and methylation. The available sources of folate supplement included biofortification by biosynthetic folate in crops (Strobbe and Van, 2017). Previously, Shan et al. (2019) reported that GTP cyclohydrolase (GTPCH) played an important role in folate biosynthesis during waxy maize grain development. In contrast, a total of three DEGs were detected among all comparison groups that controlled folate biosynthesis, including one folB and two GGH genes (**Figure 4F**). Therefore, this work also supplemented the gene resources involved in folate biosynthesis.

### Complex interaction mechanism between quality and volatile flavors during waxy maize grain formation

**Figure 5:**
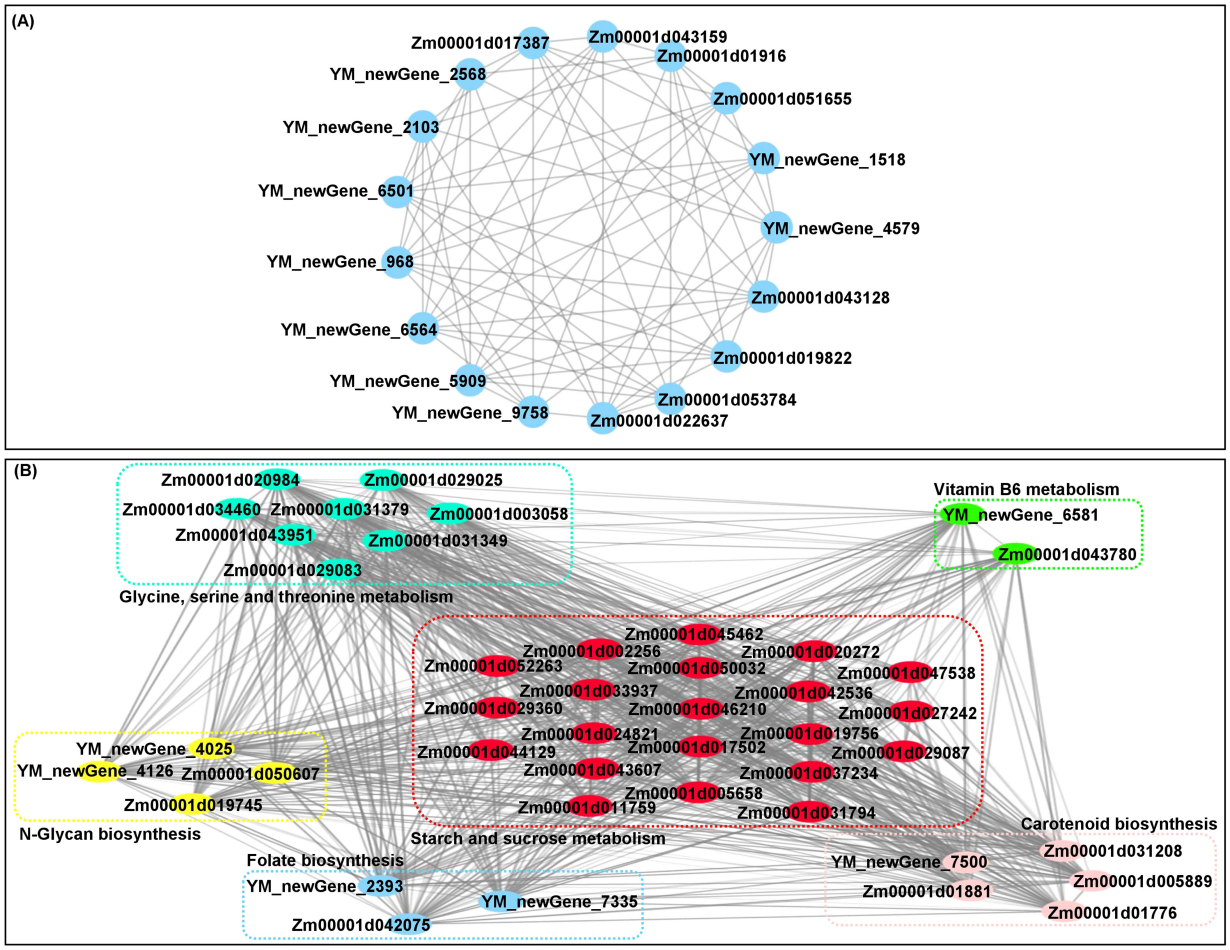
The interaction networks mapping of corresponding differentially expressed genes (DEGs) in grains of “Jingkenuo 2000” at 15 days after pollination under three N treatments (IF: 100% inorganic N fertilizer, OF: 100% organic N fertilizer, OF_IF: organic fertilizer substituting 50% inorganic N fertilizer). **(A)** Interaction networks mapping of 17 core conserved DEGs. **(B)** Interaction networks mapping of 43 unique DEGs involved in multi-nutrient formation and metabolism.

**Figure 6:**
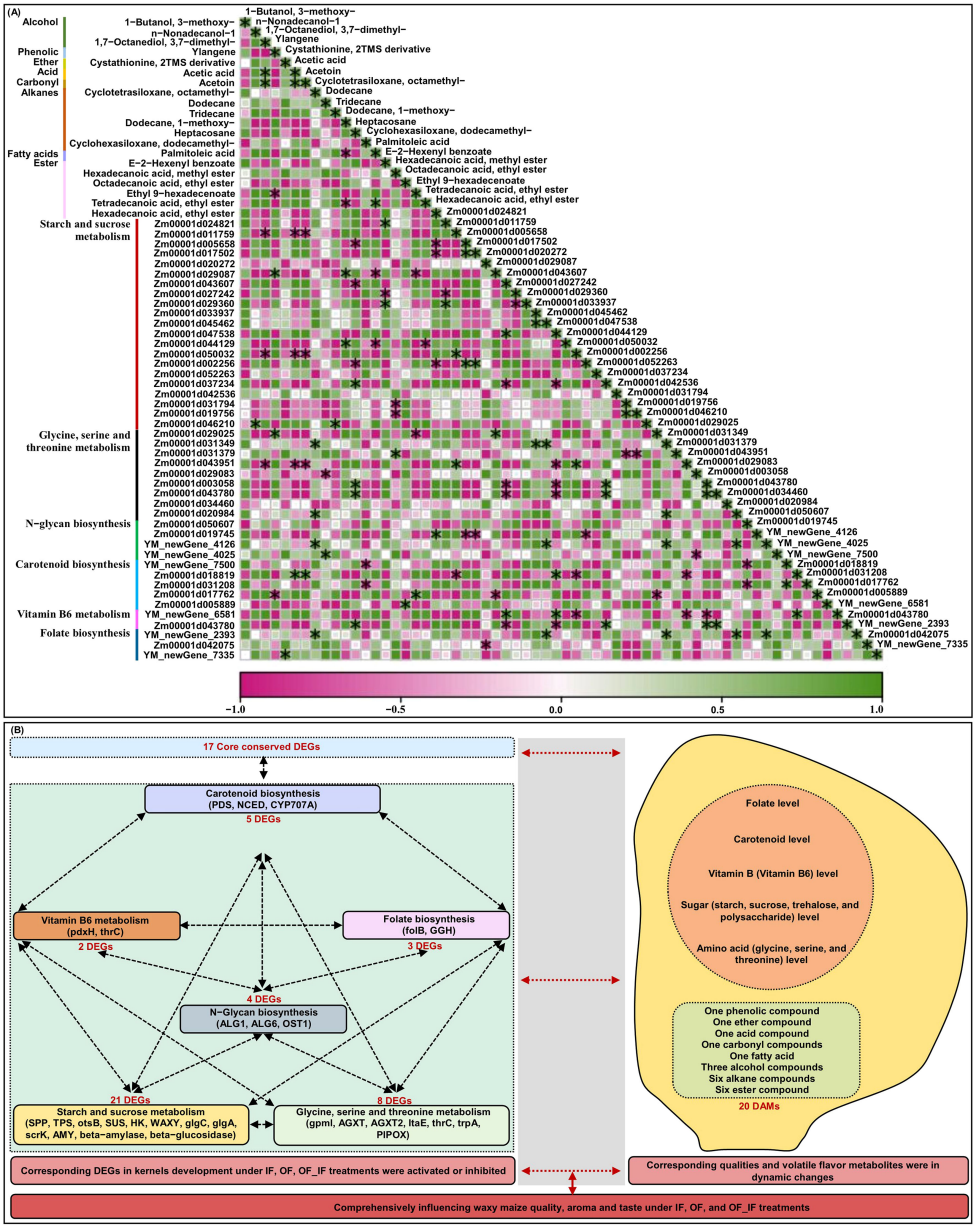
Joint Pearson correlation analysis among volatile flavors accumulation and quality-related genes expression levels in grains of “Jingkenuo 2000” at 15 days after pollination, and molecular network model underlying waxy maize grain quality and aroma volatiles formation under three N treatments (IF: 100% inorganic N fertilizer, OF: 100% organic N fertilizer, OF_IF: organic fertilizer substituting 50% inorganic N fertilizer). **(A)** Joint Pearson correlation among volatile flavors accumulation and quality related genes expression levels. * indicates a significant Pearson correlation with *p* < 0.05. **(B)** Molecular network model underlying waxy maize grain quality and aroma volatiles formation. DEGs, differentially expressed genes; DAMs, differentially accumulated metabolites.

There was a complex interaction network for multiple quality-related DEGs that involved in starch and sucrose metabolism (21), glycine, serine and threonine metabolism (9), N-glycan biosynthesis (4), carotenoid biosynthesis (5), vitamin B6 metabolism (2), and folate biosynthesis (3) in waxy maize grains under different N treatments (**Figure 5B**). The findings illustrated that the pathway networks and multiple unknown pathways involved in grain quality formation represent potential points of crosstalk. Moreover, we previously found 18 core conserved DEGs among all groups (**Figures 2E, F**). We further experimentally generated a system map of 17 core conserved DEGs networks involved in waxy maize grain development and grain quality formation among all groups (**Figure 5A**), highlighting the important candidate genes related to N response in waxy maize grain development and quality formation. Additionally, the Pearson correlation analysis predicted 118 groups with significant (*p* < 0.05) correlation between volatile DAMs accumulation by HS-SPME-GC/MS and/or quality-related DEGs FPKM expression level by RNA-Seq from grains of “Jingkenuo 2000” at DAP20 under three N treatments (**Figure 6A**). To summarize above, we speculated that the volatile flavors accumulation (i.e., three alcohol, one phenolic, one ether, one acid, one carbonyl, six alkanes, one fatty acid, and six ester compounds, as well as quality nutriments biosynthesis and metabolism including sugar, amino acids, carotenoid, vitamin B6, and folate) work together to influence the waxy maize grain quality, aroma, and taste under IF, OF, and OF_IF treatments. Meanwhile, it is necessary to select different waxy maize materials at multiple harvesting times, construct a dynamic regulatory network for waxy maize quality and aroma formation, will provide a valuable reference for waxy maize breeding.

## Conclusion

To control the total N amount, the substitution of organic fertilizer for partial inorganic N fertilizer could significantly affect waxy maize grain quality and volatile aroma formation. Here, we integrated transcriptome and volatile flavor profiles to comprehensively reveal the formation mechanism of waxy maize quality and aroma at DAP20 under IF, OF, and OF_IF treatments (**Figure 6B**). The results indicated that the esters decreased under IF treatment, alkanes increased under OF treatment, esters had the highest abundance under OF_IF treatment, and hexadecanoic acid, 1,7-octanediol, 3,7-dimethyl-, acetoin, cyclohexasiloxane, dodecamethyl-, ethyl-9-hexadecenoate, and tetradecanoic acid, were marker metabolites of waxy maize grains at OF_IF application. Moreover, a total of 43 unique candidate DEGs involved in starch and sucrose metabolism, glycine, serine and threonine metabolism, N-glycan biosynthesis, carotenoid biosynthesis, vitamin B6 metabolism, and folate biosynthesis were identified, of which high interaction networks with each other influenced multi-nutrients formation and metabolism under different N treatments. It seems that the complex quality and aroma of waxy maize under IF, OF, and OF_IF applications is the result of a combination of multiple flavor volatiles and nutrients. Therefore, the results constitute a preliminary atlas of spatiotemporal patterns of grain quality gene expression and aroma volatiles, which will support future efforts to understand the underlying mechanisms that control waxy maize grain quality and aroma formation under organic fertilizer substitution.

## Data Availability

The original contributions presented in the study are publicly available. This data can be found here: National Central for Biotechnology Information (NCBI) BioProject database under accession number PRJNA1050395.
