# Supplementary material for: Organic fertilizer substitution altered the waxy maize grain quality and aroma volatiles formation by the integrated transcriptomic and metabolomic analyses
Source: Front Plant Sci. 2025 Jun 13;16:1581728. doi: 10.3389/fpls.2025.1581728 (PMC12202232; doi:10.3389/fpls.2025.1581728)
Supplement: Supplementary file 1 [file SupplementaryFile1.docx]

**
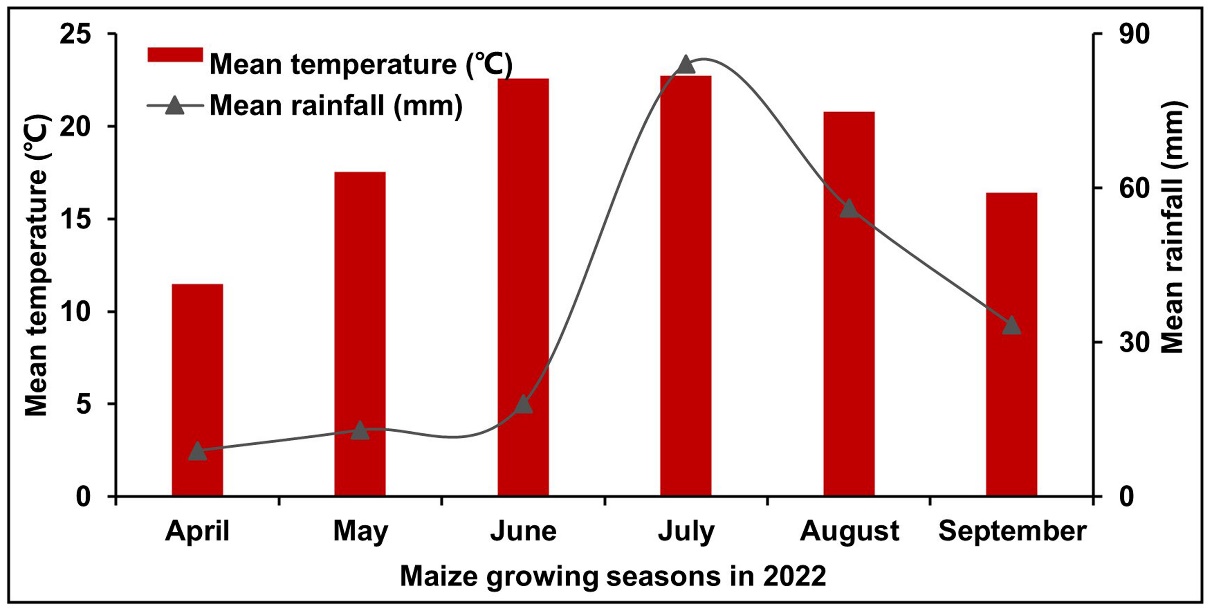
**

**Supplementary Figure 1** Weather data of maize growing seasons (from April to September) at the Oasis Agricultural Trial Station, Gansu, China in 2022.

**
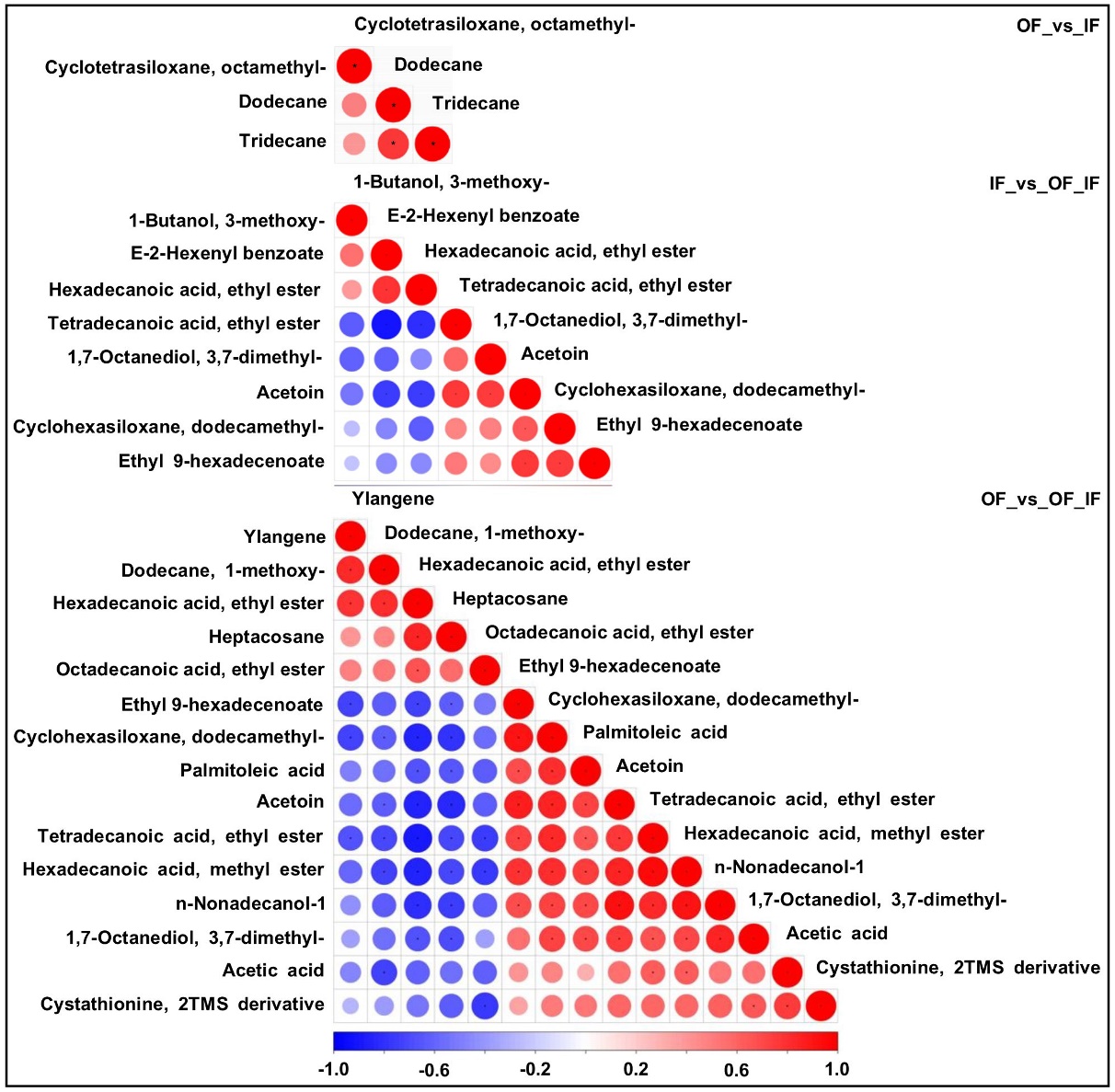
**

**Supplementary Figure 2** Pearson correlation coefficient diagrams (* indicates significant Pearson correlation with *p* < 0.05) among all differentially accumulated volatile flavors in three groups (OF_vs_IF, IF_vs_OF_IF, and OF_vs_OF_IF; IF: 100% inorganic N fertilizer, OF: 100% organic N fertilizer, OF_IF: organic fertilizer substituting 50% inorganic N fertilizer).


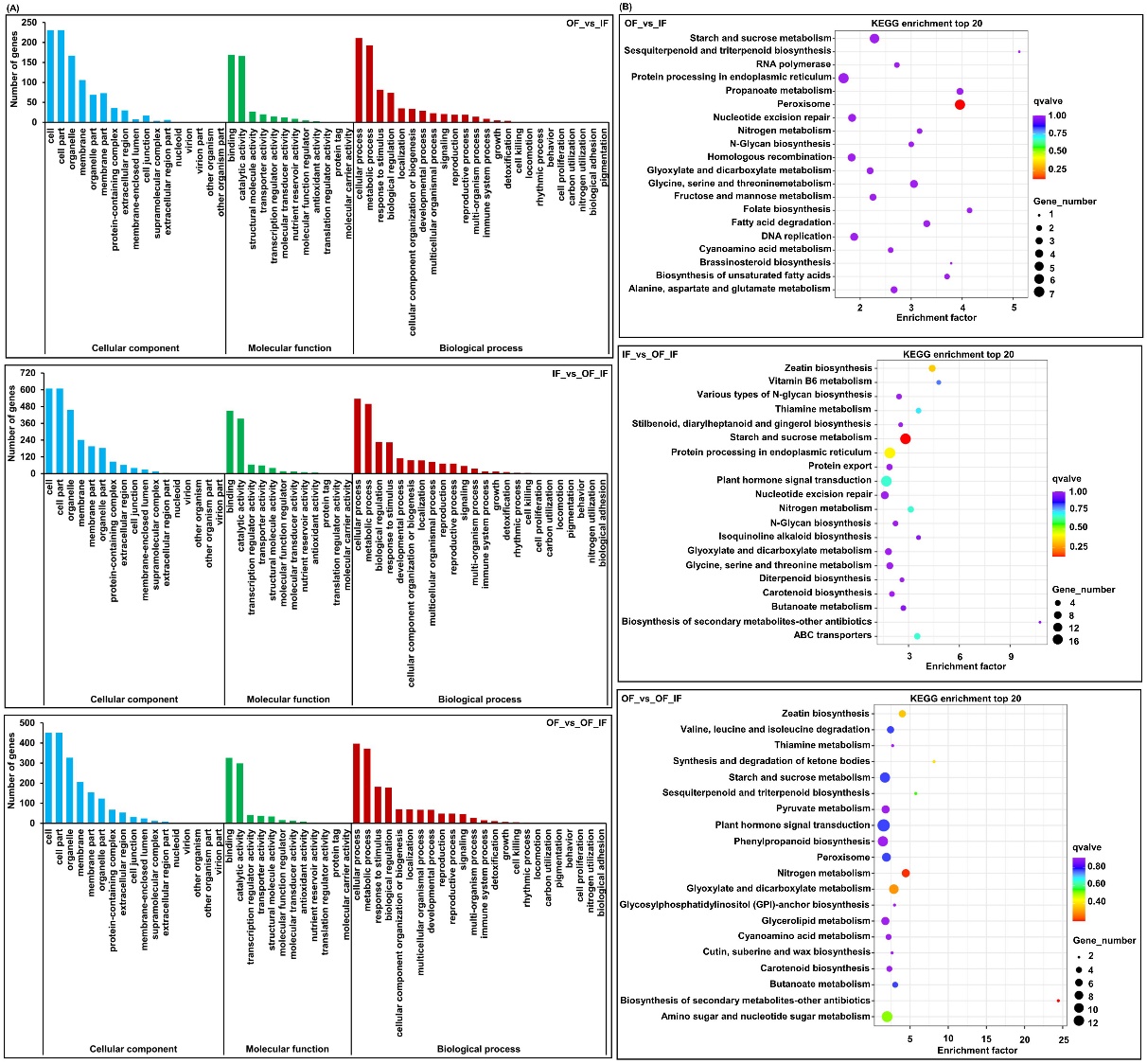


**Supplementary Figure 3** Functional annotation analyses of all differentially expressed genes (DEGs) in grains of “Jingkenuo 2000” at 15 days after pollination among three groups (IF_vs_OF_IF, OF_vs_IF, and OF_vs_OF_IF; IF: 100% inorganic N fertilizer, OF: 100% organic N fertilizer, OF_IF: organic fertilizer substituting 50% inorganic N fertilizer). (A) Enriched GO terms. (B) Top 20 KEGG pathways.
