# Supplementary material for: Organic fertilizer substitution altered the waxy maize grain quality and aroma volatiles formation by the integrated transcriptomic and metabolomic analyses
Source: Front Plant Sci. 2025 Jun 13;16:1581728. doi: 10.3389/fpls.2025.1581728 (PMC12202232; doi:10.3389/fpls.2025.1581728)
Supplement: Supplementary file 2 [file SupplementaryFile2.docx]

**Supplementary Table 1** Summary of RNA quality.

| **Sample Code** | **Concentration (ng/μL)** | **Volumme (μL)** | **Amount (μg)** | **OD_260/280_** | **OD_260/230_** |
| --- | --- | --- | --- | --- | --- |
| IF1 | 88.56 | 40 | 3.54 | 2.19 | 2.22 |
| IF2 | 163.04 | 40 | 6.52 | 2.17 | 2.18 |
| IF3 | 111.36 | 40 | 4.45 | 2.20 | 2.13 |
| OF1 | 74.71 | 40 | 2.99 | 2.18 | 2.12 |
| OF2 | 50.64 | 40 | 2.03 | 2.08 | 1.34 |
| OF3 | 147.25 | 40 | 5.89 | 2.19 | 2.25 |
| OF_IF1 | 71.52 | 40 | 2.86 | 2.18 | 1.74 |
| OF_IF2 | 134.95 | 40 | 5.40 | 2.20 | 2.28 |
| OF_IF3 | 90.54 | 40 | 3.62 | 2.19 | 2.28 |

IF: 100% inorganic N fertilizer, OF: 100% organic N fertilizer, OF_IF: organic fertilizer substituting 50% inorganic N fertilizer.

**Supplementary Table 2** Primers sequences of eight random genes used for quantitative real-time PCR (qRT-PCR) analysis.

| **Gene ID** | **Encoded protein** | **Chromosome** | **Position (bp)** | **Exon count** | **Forward primer (5' to 3')** | **Reverse primer (5' to 3')** |
| --- | --- | --- | --- | --- | --- | --- |
| *Zm00001d044129* | Shrunken 2 | 3 | 219979958..219991519 | 19 | GATGGGATTGACAGGTTGGAAA | CCGCCCAAAATGATAGCAGAT |
| *Zm00001d037234* | Soluble starch synthase 2-3 | 6 | 117124074..117128209 | 9 | TCCTGACACCGATGGTATTGC | GCATCATCTGCACCAACCCT |
| *Zm00001d003058* | Putative threonine aldolase family protein | 2 | 30708193..30711201 | 9 | TAGGCGGTGGAATGAGACAG | TTCTTCAGTCCCTCTGCCAAA |
| *Zm00001d020984* | Unknown | 7 | 139074711..139076904 | 2 | AAGCGAGGTGAAGGAGAGGG | CCAGTAGCAGATGAGCGTGTG |
| *YM_newGene_4126* | Dolichyl-diphosphooligosaccharide--protein glycosyltransferase 67 kDa subunit | 5 | 232448..2324945 | - | CTATCAGATTGCCTGGTGGGA | CAGGTAAGTAGGAGAACGGGG |
| *Zm00001d017762* | Putative cytochrome P450 superfamily protein precursor | 5 | 206138077..206140327 | 5 | GTGGCGTCCATCCTGTCCTT | TGGGAGCAACCTCGAATCG |
| *Zm00001d043780* | Threonine synthase | 3 | 210000316..210002265 | 1 | CCTTGAGGGGCAGAAGACAG | TCTCAAACCCCTTGTAGAATGC |
| *YM_newGene_7335* | MLO-like protein 4 | 7 | 142926056..142926643 | - | GAGGAAACCAATGATCCCACAC | GCAAGTTTCATCAAAGCAAGGT |
| *ZmActin1* | Actin-1 isoform X1 | 8 | 102413768..102417536 | 5 | CGATTGAGCATGGCATTGTCA | CCCACTAGCGTACAACGAA |

**Supplementary Table 3** The annotation information of 56 detected volatile flavors in grains of “Jingkenuo 2000” at 15 days after pollination under three N treatments.

| **Metabolite** | **KEGG Compount Name** | **Super.Class** | **Class** | **Sub.Class** |
| --- | --- | --- | --- | --- |
| 1,4,7,10,13,16-Hexaoxacyclooctadecane | 1,4,7,10,13,16-Hexaoxacyclooctadecane | Organic oxygen compounds | Organooxygen compounds | Ethers |
| 1,7-Octanediol, 3,7-dimethyl- |  |  |  |  |
| 15-Crown-5 |  |  |  |  |
| 1-Butanol, 3-methoxy- |  |  |  |  |
| 1-Butanol, 3-methyl- |  |  |  |  |
| 1-Undecyne |  |  |  |  |
| 2,4-Di-tert-butylphenol | 2,4-Di-tert-butylphenol | Benzenoids | Benzene and substituted derivatives | Phenylpropanes |
| 2-Heptanol |  |  |  |  |
| 2-Hexadecanol |  |  |  |  |
| 2-Methoxy-4-vinylphenol | 2-Methoxy-4-vinylphenol | Benzenoids | Phenols | Methoxyphenols |
| 2-Tridecanol |  |  |  |  |
| 3-Heptanol, 2-methyl- |  |  |  |  |
| 7-Hexadecenoic acid, methyl ester, (Z)- |  |  |  |  |
| Acetic acid | Acetylglycine | Organic acids and derivatives | Carboxylic acids and derivatives | Amino acids, peptides, and analogues |
| Acetoin | Acetoin | Organic oxygen compounds | Organooxygen compounds | Carbonyl compounds |
| Allyl 2-ethyl butyrate | 2-Propenyl 2-ethylbutanoate | Lipids and lipid-like molecules | Fatty Acyls | Fatty acid esters |
| Arginine | Arginine hydrochloride | Organic acids and derivatives | Carboxylic acids and derivatives | Amino acids, peptides, and analogues |
| Butanal, 3-hydroxy- |  |  |  |  |
| Butyrolactone | gamma-Butyrolactone | Organoheterocyclic compounds | Lactones | Gamma butyrolactones |
| cis-7-Hexadecenoic acid |  |  |  |  |
| Cycloheptasiloxane, tetradecamethyl- |  |  |  |  |
| Cyclohexasiloxane, dodecamethyl- |  |  |  |  |
| Cyclononasiloxane, octadecamethyl- |  |  |  |  |
| Cyclooctasiloxane, hexadecamethyl- |  |  |  |  |
| Cyclopentasiloxane, decamethyl- |  |  |  |  |
| Cyclotetrasiloxane, octamethyl- |  |  |  |  |
| Cystathionine, 2TMS derivative |  |  |  |  |
| d-Mannose | D-Mannose | Organic oxygen compounds | Organooxygen compounds | Carbohydrates and carbohydrate conjugates |
| Dodecane | N-Dodecane | Hydrocarbons | Saturated hydrocarbons | Alkanes |
| Dodecane, 1-methoxy- |  |  |  |  |
| E-2-Hexenyl benzoate |  |  |  |  |
| Ethanol, 2-(hexadecyloxy)- |  |  |  |  |
| Ethanol, 2-(methylamino)- |  |  |  |  |
| Ethoxy(dimethyl)isopropylsilane |  |  |  |  |
| Ethyl 9-hexadecenoate | Ethyl 9-hexadecenoate | Lipids and lipid-like molecules | Fatty Acyls | Fatty acid esters |
| Furan, 2-pentyl- |  |  |  |  |
| Heptacosane | Heptacosane | Hydrocarbons | Saturated hydrocarbons | Alkanes |
| Heptaethylene glycol | Heptaethylene glycol | Organic oxygen compounds | Organooxygen compounds | Ethers |
| Hexadecane | Hexadecane | Hydrocarbons | Saturated hydrocarbons | Alkanes |
| Hexadecanoic acid, ethyl ester | Ethyl hexadecanoate | Lipids and lipid-like molecules | Fatty Acyls | Fatty acid esters |
| Hexadecanoic acid, methyl ester |  |  |  |  |
| Methyl stearate | Methyl stearate | Lipids and lipid-like molecules | Fatty Acyls | Fatty acid esters |
| n-Nonadecanol-1 |  |  |  |  |
| Nonadecane | Nonadecane | Hydrocarbons | Saturated hydrocarbons | Alkanes |
| Octadecane, 1-(ethenyloxy)- |  |  |  |  |
| Octadecanoic acid, ethyl ester | Ethyl stearate | Lipids and lipid-like molecules | Fatty Acyls | Fatty acid esters |
| Palmitoleic acid | Palmitelaidic acid | Lipids and lipid-like molecules | Fatty Acyls | Fatty acids and conjugates |
| Tetradecane | Tetradecane | Hydrocarbons | Saturated hydrocarbons | Alkanes |
| Tetradecane, 1-chloro- |  |  |  |  |
| Tetradecanoic acid, ethyl ester | Ethyl tetradecanoate | Lipids and lipid-like molecules | Fatty Acyls | Fatty acid esters |
| trans-Sesquisabinene hydrate |  |  |  |  |
| Tridecane | Tridecane | Hydrocarbons | Saturated hydrocarbons | Alkanes |
| Tridecane, 3-methyl- |  |  |  |  |
| Ylangene | Ylangene | Lipids and lipid-like molecules | Prenol lipids | Sesquiterpenoids |
| ..-Elemene |  |  |  |  |
| 3-Heptanone, 2-methyl- |  |  |  |  |

**Supplementary Table 4** Summary of quality preprocessing of RNA-sequencing (RNA-Seq) data.

| **Sample**  **codes** | **Clean date size (bp)** | **Total reads** | **Q20 percentage (%)** | **Q30 percentage (%)** | **GC (%)** | **Mapped reads** | **Mapping rate (%)** |
| --- | --- | --- | --- | --- | --- | --- | --- |
| IF1 | 8,006,762,400 | 49,104,238 | 97.24 | 92.36 | 53.98 | 45,170,989 | 91.99 |
| IF2 | 7,887,854,100 | 48,251,076 | 96.86 | 91.57 | 53.54 | 44,275,187 | 91.76 |
| IF3 | 9,244,744,800 | 56,649,259 | 97.62 | 93.27 | 53.70 | 52,071,999 | 91.92 |
| OF1 | 8,930,632,200 | 54,635,104 | 97.11 | 91.95 | 54.03 | 50,138,635 | 91.77 |
| OF2 | 8,198,208,000 | 50,178,098 | 97.50 | 92.96 | 54.13 | 46,068,512 | 91.81 |
| OF3 | 6,532,706,100 | 40,009,616 | 97.37 | 92.69 | 53.82 | 36,756,834 | 91.87 |
| OF_IF1 | 8,197,492,800 | 49,674,150 | 96.84 | 91.49 | 53.96 | 45,153,802 | 90.90 |
| OF_IF2 | 7,421,565,900 | 45,592,476 | 97.30 | 92.54 | 53.81 | 42,013,467 | 92.15 |
| OF_IF3 | 8,060,241,900 | 48,490,602 | 97.07 | 92.10 | 54.33 | 43,757,919 | 90.24 |

IF: 100% inorganic N fertilizer, OF: 100% organic N fertilizer, OF_IF: organic fertilizer substituting 50% inorganic N fertilizer.

**Supplementary Table 5** Expression profiles and function annotations of 18 core conserved differentially expressed genes (DEGs) in three groups.

| **Gene_ID** | **log2 Fold-Change** | | | **GO_annotation Nr_annotation** | |
| --- | --- | --- | --- | --- | --- |
|  | **OF_vs_IF** | **IF_vs_OF_IF** | **OF_vs_OF_IF** |  |  |
| YM_newGene_10261 | 2.03 | -4.22 | -2.19 | -- | Succinate dehydrogenase assembly factor 1A |
| YM_newGene_1518 | 2.10 | 1.71 | 3.81 | -- | Zinc finger MYM-type protein 1-like |
| YM_newGene_2103 | 5.45 | 1.61 | 7.05 | Molecular Function: DNA binding (GO:0003677);; Biological Process: DNA recombination (GO:0006310);; Biological Process: transposition (GO:0032196);; | hAT family dimerisation domain containing protein |
| YM_newGene_2568 | 4.47 | 1.81 | 6.28 | Molecular Function: phosphoprotein phosphatase activity (GO:0004721);; Cellular Component: nucleus (GO:0005634);; Molecular Function: metal ion binding (GO:0046872);; | Transposase related protein |
| YM_newGene_4579 | 2.17 | -3.69 | -1.52 | Molecular Function: structural constituent of cell wall (GO:0005199);; | Hypothetical protein |
| YM_newGene_5909 | 1.62 | -6.18 | -4.56 | -- | Unknown |
| YM_newGene_6501 | -2.27 | 3.53 | 1.27 | Molecular Function: thiol-dependent ubiquitin-specific protease activity (GO:0004843);; Biological Process: ubiquitin-dependent protein catabolic process (GO:0006511);; Biological Process: protein deubiquitination (GO:0016579);; | Hypothetical protein |
| YM_newGene_6564 | -2.86 | 5.44 | 2.58 | Cellular Component: nucleus (GO:0005634);; Biological Process: regulation of transcription, DNA-templated (GO:0006355);; Molecular Function: sequence-specific DNA binding (GO:0043565);; | Serine/arginine repetitive matrix protein 1-like |
| YM_newGene_9680 | 1.22 | -2.93 | -1.70 | -- | Hypothetical protein |
| YM_newGene_9758 | 3.64 | 2.45 | 6.11 | Molecular Function: phosphoprotein phosphatase activity (GO:0004721);; Cellular Component: nucleus (GO:0005634);; Molecular Function: metal ion binding (GO:0046872);; | Uncharacterized protein LOC1003042 |
| Zm00001d017387 | 1.16 | -2.38 | -1.23 | Biological Process: resolution of meiotic recombination intermediates (GO:0000712);; Cellular Component: nucleus (GO:0005634);; Cellular Component: plasmodesma (GO:0009506);; | Shortage in chiasmata 1 |
| Zm00001d019162 | 1.40 | -5.65 | -4.25 | Molecular Function: nutrient reservoir activity (GO:0045735);; | Hypothetical protein |
| Zm00001d019822 | 1.99 | 1.57 | 3.56 | -- | ARM repeat superfamily protein |
| Zm00001d022637 | 2.16 | -3.49 | -1.32 | -- | thaumatin-like protein 1 |
| Zm00001d043128 | 1.07 | 1.22 | 2.30 | Cellular Component: plasma membrane (GO:0005886);; Biological Process: defense response (GO:0006952);; Cellular Component: plasmodesma (GO:0009506);; Cellular Component: integral component of membrane (GO:0016021);; | Unknown |
| Zm00001d043159 | 3.96 | 1.31 | 5.28 | Cellular Component: nucleus (GO:0005634);; Biological Process: regulation of transcription, DNA-templated (GO:0006355);; Biological Process: response to cold (GO:0009409);; Biological Process: leaf senescence (GO:0010150);; Biological Process: defense response to bacterium (GO:0042742);; Molecular Function: sequence-specific DNA binding (GO:0043565);; Biological Process: positive regulation of transcription by RNA polymerase II (GO:0045944);; Biological Process: defense response to fungus (GO:0050832);; | Uncharacterized protein LOC100383685 |
| Zm00001d051655 | 1.87 | 1.50 | 3.38 | -- | Hypothetical protein |
| Zm00001d053784 | -1.05 | -1.03 | -2.07 | Cellular Component: extracellular region (GO:0005576);; Biological Process: killing of cells of other organism (GO:0031640);; Biological Process: defense response to fungus (GO:0050832);; | Gamma-thionins family protein precursor |

IF: 100% inorganic N fertilizer, OF: 100% organic N fertilizer, OF_IF: organic fertilizer substituting 50% inorganic N fertilizer.

**Supplementary Table 6** Function annotation of 21 differentially expressed genes (DEGs) involved in starch and sucrose metabolism.

| **Gene_ID** | **GO_annotation** | **Nr_annotation** |
| --- | --- | --- |
| Zm00001d020272 | Molecular Function: trehalose-phosphatase activity (GO:0004805);; Biological Process: trehalose biosynthetic process (GO:0005992);; | Probable trehalose-phosphate phosphatase 7 isoform X5 |
| Zm00001d045462 | Molecular Function: glycogen (starch) synthase activity (GO:0004373);; Cellular Component: amyloplast (GO:0009501);; Cellular Component: chloroplast (GO:0009507);; Biological Process: starch biosynthetic process (GO:0019252);; Molecular Function: NDP-glucose-starch glucosyltransferase activity (GO:0033840);; | Granule-bound starch synthase 1 |
| Zm00001d027242 | Molecular Function: glycogen (starch) synthase activity (GO:0004373);; Cellular Component: amyloplast (GO:0009501);; Cellular Component: chloroplast (GO:0009507);; Biological Process: starch biosynthetic process (GO:0019252);; Molecular Function: NDP-glucose-starch glucosyltransferase activity (GO:0033840);; | Granule-bound starch synthase 1 |
| Zm00001d042536 | Molecular Function: ribokinase activity (GO:0004747);; Molecular Function: ATP binding (GO:0005524);; Biological Process: D-ribose metabolic process (GO:0006014);; Molecular Function: fructokinase activity (GO:0008865);; Biological Process: starch biosynthetic process (GO:0019252);; | Fructokinase1 |
| Zm00001d031794 | Molecular Function: alpha-amylase activity (GO:0004556);; Molecular Function: calcium ion binding (GO:0005509);; Biological Process: starch catabolic process (GO:0005983);; Biological Process: sucrose catabolic process (GO:0005987);; | Putative alpha-amylase family protein precursor |
| Zm00001d019756 | Biological Process: polysaccharide catabolic process (GO:0000272);; Cellular Component: chloroplast (GO:0009507);; Cellular Component: chloroplast stroma (GO:0009570);; Molecular Function: beta-amylase activity (GO:0016161);; | Beta-amylase |
| Zm00001d024821 | Molecular Function: magnesium ion binding (GO:0000287);; Biological Process: sucrose biosynthetic process (GO:0005986);; Molecular Function: sucrose-phosphate phosphatase activity (GO:0050307);; | Sucrose-phosphatase 2 |
| Zm00001d011759 | Cellular Component: cytosol (GO:0005829);; Biological Process: trehalose biosynthetic process (GO:0005992);; Molecular Function: transferase activity, transferring glycosyl groups (GO:0016757);; Molecular Function: phosphatase activity (GO:0016791);; Biological Process: trehalose metabolism in response to stress (GO:0070413);; | Probable alpha,alpha-trehalose-phosphate synthase [UDP-forming] 7 |
| Zm00001d005658 | Molecular Function: trehalose-phosphatase activity (GO:0004805);; Biological Process: trehalose biosynthetic process (GO:0005992);; | Unknown |
| Zm00001d017502 | Molecular Function: trehalose-phosphatase activity (GO:0004805);; Biological Process: trehalose biosynthetic process (GO:0005992);; Biological Process: response to cold (GO:0009409);; Biological Process: response to salt stress (GO:0009651);; | Trehalose-6-phosphate phosphatase6 |
| Zm00001d029087 | Biological Process: sucrose metabolic process (GO:0005985);; Molecular Function: sucrose synthase activity (GO:0016157);; | Sucrose synthase 3 |
| Zm00001d043607 | Biological Process: cellular glucose homeostasis (GO:0001678);; Molecular Function: glucokinase activity (GO:0004340);; Molecular Function: ATP binding (GO:0005524);; Molecular Function: glucose binding (GO:0005536);; Cellular Component: cytosol (GO:0005829);; Biological Process: glycolytic process (GO:0006096);; Molecular Function: fructokinase activity (GO:0008865);; Molecular Function: mannokinase activity (GO:0019158);; | Uncharacterized protein LOC100279587 |
| Zm00001d029360 | Molecular Function: glycogen (starch) synthase activity (GO:0004373);; Cellular Component: amyloplast (GO:0009501);; Cellular Component: chloroplast (GO:0009507);; Biological Process: starch biosynthetic process (GO:0019252);; Molecular Function: NDP-glucose-starch glucosyltransferase activity (GO:0033840);; | Granule-bound starch synthase 1 |
| Zm00001d033937 | Molecular Function: glycogen (starch) synthase activity (GO:0004373);; Cellular Component: amyloplast (GO:0009501);; Cellular Component: chloroplast (GO:0009507);; Biological Process: starch biosynthetic process (GO:0019252);; Molecular Function: NDP-glucose-starch glucosyltransferase activity (GO:0033840);; | Granule-bound starch synthase 1 |
| Zm00001d047538 | Molecular Function: cellulase activity (GO:0008810);; Cellular Component: integral component of membrane (GO:0016021);; Biological Process: cellulose catabolic process (GO:0030245);; Biological Process: cell wall organization (GO:0071555);; | Endo-1,4-beta-glucanase |
| Zm00001d002256 | Molecular Function: glycogen (starch) synthase activity (GO:0004373);; Molecular Function: starch synthase activity (GO:0009011);; Cellular Component: amyloplast (GO:0009501);; Cellular Component: chloroplast (GO:0009507);; Biological Process: starch biosynthetic process (GO:0019252);; Molecular Function: alpha-1,4-glucan synthase activity (GO:0033201);; Molecular Function: starch binding (GO:2001070);; | Starch synthase 3 |
| Zm00001d052263 | Molecular Function: glycogen (starch) synthase activity (GO:0004373);; Molecular Function: starch synthase activity (GO:0009011);; Cellular Component: amyloplast (GO:0009501);; Cellular Component: chloroplast (GO:0009507);; Biological Process: starch biosynthetic process (GO:0019252);; Molecular Function: alpha-1,4-glucan synthase activity (GO:0033201);; | Starch synthase 2 |
| Zm00001d037234 | Molecular Function: glycogen (starch) synthase activity (GO:0004373);; Molecular Function: starch synthase activity (GO:0009011);; Cellular Component: amyloplast (GO:0009501);; Cellular Component: chloroplast (GO:0009507);; Biological Process: starch biosynthetic process (GO:0019252);; Molecular Function: alpha-1,4-glucan synthase activity (GO:0033201);; | Sugary2 |
| Zm00001d046210 | Biological Process: carbohydrate metabolic process (GO:0005975);; Molecular Function: beta-glucosidase activity (GO:0008422);; Molecular Function: scopolin beta-glucosidase activity (GO:0102483);; Biological Process: glycosyl compound metabolic process (GO:1901657);; | Beta-glucosidase 11 |
| Zm00001d044129 | Molecular Function: ATP binding (GO:0005524);; Biological Process: glycogen biosynthetic process (GO:0005978);; Molecular Function: glucose-1-phosphate adenylyltransferase activity (GO:0008878);; Cellular Component: amyloplast (GO:0009501);; Cellular Component: chloroplast (GO:0009507);; Biological Process: starch biosynthetic process (GO:0019252);; | Glucose-1-phosphate adenylyltransferase large subunit 1 |
| Zm00001d050032 | Molecular Function: ATP binding (GO:0005524);; Biological Process: glycogen biosynthetic process (GO:0005978);; Biological Process: starch metabolic process (GO:0005982);; Molecular Function: glucose-1-phosphate adenylyltransferase activity (GO:0008878);; Cellular Component: amyloplast (GO:0009501);; Cellular Component: chloroplast (GO:0009507);; Biological Process: starch biosynthetic process (GO:0019252);; | Glucose-1-phosphate adenylyltransferase |

IF: 100% inorganic N fertilizer, OF: 100% organic N fertilizer, OF_IF: organic fertilizer substituting 50% inorganic N fertilizer.

**Supplementary Table 7** Function annotation of nine differentially expressed genes (DEGs) involved in glycine, serine and threonine metabolism.

| **Gene_ID** | **GO_annotation** | **Nr_annotation** |
| --- | --- | --- |
| Zm00001d031349 | Molecular Function: serine-pyruvate transaminase activity (GO:0004760);; Cellular Component: peroxisome (GO:0005777);; Cellular Component: plasma membrane (GO:0005886);; Molecular Function: alanine-glyoxylate transaminase activity (GO:0008453);; Cellular Component: chloroplast (GO:0009507);; Cellular Component: chloroplast stroma (GO:0009570);; Biological Process: photorespiration (GO:0009853);; Cellular Component: membrane (GO:0016020);; Biological Process: glycine biosynthetic process, by transamination of glyoxylate (GO:0019265);; Cellular Component: apoplast (GO:0048046);; Molecular Function: serine-glyoxylate transaminase activity (GO:0050281);; | Uncharacterized protein LOC100281949 |
| Zm00001d034460 | Biological Process: tryptophan biosynthetic process (GO:0000162);; Molecular Function: tryptophan synthase activity (GO:0004834);; Cellular Component: chloroplast (GO:0009507);; Cellular Component: chloroplast stroma (GO:0009570);; Biological Process: gravitropism (GO:0009630);; Biological Process: response to cytokinin (GO:0009735);; Biological Process: auxin biosynthetic process (GO:0009851);; Molecular Function: indole-3-glycerol-phosphate lyase activity (GO:0033984);; Biological Process: defense response to bacterium (GO:0042742);; Biological Process: defense response by callose deposition in cell wall (GO:0052544);; | Indole-3-glycerol phosphate lyase 1 |
| Zm00001d029083 | Cellular Component: mitochondrion (GO:0005739);; Molecular Function: alanine-glyoxylate transaminase activity (GO:0008453);; Biological Process: photorespiration (GO:0009853);; Molecular Function: pyridoxal phosphate binding (GO:0030170);; Molecular Function: identical protein binding (GO:0042802);; Biological Process: cellular response to nitrogen levels (GO:0043562);; | Alanine--glyoxylate aminotransferase1 |
| Zm00001d020984 | Molecular Function: sarcosine oxidase activity (GO:0008115);; Biological Process: tetrahydrofolate metabolic process (GO:0046653);; | Unknown |
| Zm00001d029025 | Molecular Function: phosphoglycerate mutase activity (GO:0004619);; Cellular Component: cytoplasm (GO:0005737);; Biological Process: glucose catabolic process (GO:0006007);; Biological Process: glycolytic process (GO:0006096);; Molecular Function: manganese ion binding (GO:0030145);; | 2,3-bisphosphoglycerate-independent phosphoglycerate mutase isoform X1 |
| Zm00001d043951 | Cellular Component: mitochondrion (GO:0005739);; Molecular Function: alanine-glyoxylate transaminase activity (GO:0008453);; Biological Process: photorespiration (GO:0009853);; Molecular Function: pyridoxal phosphate binding (GO:0030170);; Molecular Function: identical protein binding (GO:0042802);; | Alanine--glyoxylate aminotransferase 2 homolog 2 mitochondrial |
| Zm00001d043780 | Molecular Function: threonine synthase activity (GO:0004795);; Biological Process: threonine biosynthetic process (GO:0009088);; Cellular Component: chloroplast (GO:0009507);; Molecular Function: pyridoxal phosphate binding (GO:0030170);; | Threonine synthase |
| Zm00001d031379 | Molecular Function: serine-pyruvate transaminase activity (GO:0004760);; Cellular Component: peroxisome (GO:0005777);; Cellular Component: plasma membrane (GO:0005886);; Molecular Function: alanine-glyoxylate transaminase activity (GO:0008453);; Cellular Component: chloroplast (GO:0009507);; Cellular Component: chloroplast stroma (GO:0009570);; Biological Process: photorespiration (GO:0009853);; Cellular Component: membrane (GO:0016020);; Biological Process: glycine biosynthetic process, by transamination of glyoxylate (GO:0019265);; Cellular Component: apoplast (GO:0048046);; Molecular Function: serine-glyoxylate transaminase activity (GO:0050281);; | Serine--glyoxylate aminotransferase |
| Zm00001d003058 | Molecular Function: threonine aldolase activity (GO:0004793);; Cellular Component: cytosol (GO:0005829);; Biological Process: glycine biosynthetic process (GO:0006545);; Biological Process: threonine catabolic process (GO:0006567);; Molecular Function: L-allo-threonine aldolase activity (GO:0008732);; | Putative threonine aldolase family protein |

IF: 100% inorganic N fertilizer, OF: 100% organic N fertilizer, OF_IF: organic fertilizer substituting 50% inorganic N fertilizer.

**Supplementary Table 8** Function annotation of four differentially expressed genes (DEGs) involved in N-glycan biosynthesis.

| **Gene_ID** | **GO_annotation** | **Nr_annotation** | |
| --- | --- | --- | --- |
| YM_newGene_4126 | Molecular Function: dolichyl-diphosphooligosaccharide-protein glycotransferase activity (GO:0004579);; Cellular Component: oligosaccharyltransferase complex (GO:0008250);; Cellular Component: integral component of membrane (GO:0016021);; Biological Process: protein N-linked glycosylation via asparagine (GO:0018279);; | | Dolichyl-diphosphooligosaccharide--protein glycosyltransferase 67 kDasubunit |
| Zm00001d050607 | Cellular Component: endoplasmic reticulum membrane (GO:0005789);; Biological Process: protein glycosylation (GO:0006486);; Cellular Component: integral component of membrane (GO:0016021);; Molecular Function: transferase activity, transferring glycosyl groups (GO:0016757);; Biological Process: pollen tube development (GO:0048868);; | | UDP-glycosyltransferase TURAN |
| Zm00001d019745 | Cellular Component: endoplasmic reticulum membrane (GO:0005789);; Biological Process: protein glycosylation (GO:0006486);; Cellular Component: integral component of membrane (GO:0016021);; Molecular Function: transferase activity, transferring glycosyl groups (GO:0016757);; Biological Process: pollen tube development (GO:0048868);; | | UDP-glycosyltransferase TURAN |
| YM_newGene_4025 | Cellular Component: endoplasmic reticulum membrane (GO:0005789);; Biological Process: protein N-linked glycosylation (GO:0006487);; Biological Process: oligosaccharide-lipid intermediate biosynthetic process (GO:0006490);; Cellular Component: integral component of membrane (GO:0016021);; Molecular Function: dolichyl pyrophosphate Man9GlcNAc2 alpha-1,3-glucosyltransferase activity (GO:0042281);; | | Putative dolichyl pyrophosphate Man9GlcNAc2 alpha-13-glucosyltransferase |

IF: 100% inorganic N fertilizer, OF: 100% organic N fertilizer, OF_IF: organic fertilizer substituting 50% inorganic N fertilizer.

**Supplementary Table 9** Function annotation of five differentially expressed genes (DEGs) involved in carotenoid biosynthesis.

| **Gene_ID** | **GO_annotation** | **Nr_annotation** |
| --- | --- | --- |
| YM_newGene_7500 | Cellular Component: chloroplast (GO:0009507);; Cellular Component: chromoplast (GO:0009509);; Biological Process: carotenoid biosynthetic process (GO:0016117);; Molecular Function: oxidoreductase activity, acting on paired donors, with incorporation or reduction of molecular oxygen (GO:0016705);; | 15-cis-phytoene desaturase chloroplastic/chromoplastic |
| Zm00001d018819 | -- | Viviparous-14 |
| Zm00001d031208 | Molecular Function: iron ion binding (GO:0005506);; Cellular Component: endoplasmic reticulum membrane (GO:0005789);; Biological Process: multicellular organism development (GO:0007275);; Biological Process: abscisic acid metabolic process (GO:0009687);; Biological Process: brassinosteroid homeostasis (GO:0010268);; Molecular Function: (+)-abscisic acid 8'-hydroxylase activity (GO:0010295);; Cellular Component: integral component of membrane (GO:0016021);; Biological Process: sterol metabolic process (GO:0016125);; Biological Process: brassinosteroid biosynthetic process (GO:0016132);; Molecular Function: oxidoreductase activity, acting on paired donors, with incorporation or reduction of molecular oxygen, NAD(P)H as one donor, and incorporation of one atom of oxygen (GO:0016709);; Molecular Function: heme binding (GO:0020037);; Biological Process: abscisic acid catabolic process (GO:0046345);; | Cytochrome P450 family protein |
| Zm00001d017762 | Molecular Function: iron ion binding (GO:0005506);; Cellular Component: endoplasmic reticulum membrane (GO:0005789);; Biological Process: multicellular organism development (GO:0007275);; Biological Process: abscisic acid metabolic process (GO:0009687);; Biological Process: brassinosteroid homeostasis (GO:0010268);; Molecular Function: (+)-abscisic acid 8'-hydroxylase activity (GO:0010295);; Cellular Component: integral component of membrane (GO:0016021);; Biological Process: sterol metabolic process (GO:0016125);; Biological Process: brassinosteroid biosynthetic process (GO:0016132);; Molecular Function: oxidoreductase activity, acting on paired donors, with incorporation or reduction of molecular oxygen, NAD(P)H as one donor, and incorporation of one atom of oxygen (GO:0016709);; Molecular Function: heme binding (GO:0020037);; Biological Process: abscisic acid catabolic process (GO:0046345);; | Putative cytochrome P450 superfamily protein precursor |
| Zm00001d005889 | Molecular Function: iron ion binding (GO:0005506);; Biological Process: multicellular organism development (GO:0007275);; Biological Process: brassinosteroid homeostasis (GO:0010268);; Molecular Function: (+)-abscisic acid 8'-hydroxylase activity (GO:0010295);; Cellular Component: integral component of membrane (GO:0016021);; Biological Process: sterol metabolic process (GO:0016125);; Biological Process: brassinosteroid biosynthetic process (GO:0016132);; Molecular Function: heme binding (GO:0020037);; Biological Process: abscisic acid catabolic process (GO:0046345);; | Abscisic acid 8&apos;-Hydroxylase5 |

IF: 100% inorganic N fertilizer, OF: 100% organic N fertilizer, OF_IF: organic fertilizer substituting 50% inorganic N fertilizer.

**Supplementary Table 10** Function annotation of two differentially expressed genes (DEGs) involved in vitamin B6 metabolism.

| **Gene_ID** | **GO_annotation** | **Nr_annotation** |
| --- | --- | --- |
| Zm00001d043780 | Molecular Function: threonine synthase activity (GO:0004795);; Biological Process: threonine biosynthetic process (GO:0009088);; Cellular Component: chloroplast (GO:0009507);; Molecular Function: pyridoxal phosphate binding (GO:0030170);; | Threonine synthase |
| YM_newGene_6581 | Cellular Component: cytoplasm (GO:0005737);; Biological Process: actin filament organization (GO:0007015);; Cellular Component: actin filament bundle (GO:0032432);; Biological Process: actin filament severing (GO:0051014);; Molecular Function: actin filament binding (GO:0051015);; Biological Process: actin filament bundle assembly (GO:0051017);; Biological Process: actin filament capping (GO:0051693);; | Villin-4 |

IF: 100% inorganic N fertilizer, OF: 100% organic N fertilizer, OF_IF: organic fertilizer substituting 50% inorganic N fertilizer.

**Supplementary Table S11** Function annotation of three differentially expressed genes (DEGs) involved in folate biosynthesis.

| **Gene_ID** | **GO_annotation** | **Nr_annotation** |
| --- | --- | --- |
| YM_newGene_2393 | Cellular Component: endoplasmic reticulum membrane (GO:0005789);; Cellular Component: plasma membrane (GO:0005886);; Biological Process: response to abscisic acid (GO:0009737);; Molecular Function: abscisic acid binding (GO:0010427);; Cellular Component: integral component of membrane (GO:0016021);; Molecular Function: GTPase binding (GO:0051020);; Biological Process: cellular response to cold (GO:0070417);; | Hypothetical protein |
| YM_newGene_7335 | Cellular Component: extracellular space (GO:0005615);; Cellular Component: cell wall (GO:0005618);; Cellular Component: vacuole (GO:0005773);; Biological Process: glutamine metabolic process (GO:0006541);; Molecular Function: omega peptidase activity (GO:0008242);; Molecular Function: gamma-glutamyl-peptidase activity (GO:0034722);; Biological Process: tetrahydrofolylpolyglutamate metabolic process (GO:0046900);; | MLO-like protein 4 |
| Zm00001d042075 | -- | Hypothetical protein |

IF: 100% inorganic N fertilizer, OF: 100% organic N fertilizer, OF_IF: organic fertilizer substituting 50% inorganic N fertilizer.
